# Understanding the relationship between the 32-item motor function measure and daily activities from an individual with spinal muscular atrophy and their caregivers’ perspective: a two-part study

**DOI:** 10.1186/s12883-021-02166-z

**Published:** 2021-03-31

**Authors:** Tina Duong, Jessica Braid, Hannah Staunton, Aurelie Barriere, Fani Petridis, Johannes Reithinger, Rosangel Cruz, Jill Jarecki, Mencia De Lemus, Nicole Gusset, Ria Broekgaarden, Sharan Randhawa, Jessica Flynn, Rob Arbuckle, Sonia Reif, Lida Yang, Angela De Martini, Carole Vuillerot

**Affiliations:** 1 Department of Neurology and Neurological Sciences, Stanford University, Stanford, CA USA; 2 Roche Products Limited, Welwyn Garden City, UK; 3 Department of Pediatric Physical Medicine and Rehabilitation, Hôpital Mère Enfant, CHU-Lyon, Lyon University, Lyon, France; 4 F. Hoffmann-La Roche Ltd, Basel, Switzerland; 5 CureSMA, Elk Grove Village, IL USA; 6 SMA Europe Freiburg, Freiburg, Germany; 7 FundAME, Madrid, Spain; 8 SMA Schweiz, Swiss Patient Organisation for Spinal Muscular Atrophy, Heimberg, Switzerland; 9 SMA Europe and Vereniging Spierziekten Nederland, Baarn, The Netherlands; 10 Adelphi Values, Patient-Centered Outcomes, Adelphi Mill, Bollington, Cheshire UK; 11 Charles River Associates Inc, Zurich, Switzerland; 12 Neuromyogen Institute, CNRS UMR 5310 – INSERM U1217, Université de Lyon, Lyon, France

**Keywords:** Spinal muscular atrophy, 32-item motor function measure, Content validity, Qualitative interviews, Quantitative online survey, Clinical meaningfulness, Patient relevance

## Abstract

**Background:**

The 32-item Motor Function Measure (MFM32) is a clinician-reported outcome measure used to assess the functional abilities of individuals with neuromuscular diseases, including those with spinal muscular atrophy (SMA). This two-part study explored the relationship between the functional abilities assessed in the MFM32 and activities of daily living (ADLs) from the perspective of individuals with Type 2 and Type 3 (non-ambulant and ambulant) SMA and their caregivers through qualitative interviews and a quantitative online survey.

**Methods:**

In-depth, semi-structured, qualitative interviews were conducted with individuals with SMA and caregivers from the US. Subsequently, a quantitative online survey was completed by individuals with SMA or their caregivers from France, Germany, Italy, Poland, Spain, Canada, the United States (US) and the UK. In both parts of the study, participants were asked to describe the ADLs considered to be related to the functional abilities assessed in the MFM32. Results from the qualitative interviews informed the content of the quantitative online survey.

**Results:**

Qualitative interviews were conducted with 15 adult participants, and 217 participants completed the quantitative online survey. From the qualitative interviews, all of the functional abilities assessed in the patient-friendly MFM32 were deemed as related to one or more ADL. The specific ADLs that participants considered related to the patient-friendly MFM32 items could be grouped into 10 key ADL domains: dressing, mobility/transferring, self-care, self-feeding, reaching, picking up and holding objects, physical activity, writing and technology use, social contact/engagement, toileting and performing work/school activities. These results were confirmed by the quantitative online survey whereby the ADLs reported to be related to each patient-friendly MFM32 item were consistent and could be grouped into the same 10 ADL domains.

**Conclusion:**

This study provides in-depth evidence from the patient/caregiver perspective supporting the relevance of the patient-friendly MFM32 items to the ADLs of individuals with Type 2 and Type 3 SMA.

**Supplementary Information:**

The online version contains supplementary material available at 10.1186/s12883-021-02166-z.

## Background

Spinal muscular atrophy (SMA) is a rare neurodegenerative disease caused by a loss of function, mutation or deletion of the survival of motor neuron 1 gene (*SMN1*). This loss results in progressive degeneration of motor neurons in the spinal cord with atrophy of skeletal muscles and generalized muscular weakness [1]. Classically, SMA has been stratified into distinct classifications based on the age of onset and the maximum motor function achieved [2]. The three main classifications of SMA are Type 1 (Werdnig-Hoffmann disease), Type 2 (Dubowitz disease) and Type 3 (Kugelberg-Welander disease) [2, 3]. Type 1 SMA presents in the first 6 months of life and is the most severe of the three main types; these babies cannot sit without support and rarely live beyond their first years of life without treatment [2]. This study focused on individuals with Type 2 and Type 3 (non-ambulant and ambulant) SMA . Among these individuals, symptom presentation is more heterogenous and the disease is less severe than that of Type 1 SMA. Individuals with Type 2 SMA are able to sit unsupported, though they are typically unable to stand or walk independently; whereas, individuals with Type 3 SMA are able to stand and walk independently, though these abilities may be lost as the disease progresses [2, 4]. Despite this distinct subtype classification, there is often a high degree of within-group variation and thus classification is not a strict predictor of disease progression. Due to this variation, as well as the advent of approved disease-modifying therapies, classification of SMA by subtypes has evolved to focus on the functional status of the individuals (non-sitters, sitters/standers, walkers) [5, 6].

Despite approved treatments, SMA can still significantly impact an individual’s functional abilities and thus their activities of daily living (ADL) and health-related quality of life (HRQoL) [7–9]. A range of different Clinical Outcome Assessments (COAs), such as the 32-item Motor Function Measure (MFM32) [10], the Hammersmith Functional Motor Scale Expanded (HFMSE) [11] and the Revised Upper Limb Module (RULM) [12], are often used in clinical practice and as endpoints in clinical trials to assess the motor function abilities of individuals with SMA. The MFM32 is a clinician-reported outcome measure, developed based on expert clinical input to ensure the ecological validity of the items, which assesses the motor function abilities of individuals with neuromuscular disease. It has been validated for use in individuals with Type 2 and Type 3 SMA aged 2–60 years [10, 13, 14].

The MFM32 has demonstrated strong evidence of reliability, validity and responsiveness in the Type 2 and 3 SMA population [13]. However, for regulators and Health Technology Assessment (HTA) bodies [15–17], ensuring relevance of the concepts assessed from a patient perspective is an important step in the validation process. The latest Patient-Focused Drug Development guidance from the US Food and Drug Administration specifically highlights how COAs should validly and reliably measure concepts that are clinically relevant and important to patients to determine they are fit-for-purpose as endpoints in clinical trials [15, 16].

This paper describes a two-part study which aimed to further establish the relationship between the functional abilities assessed by the MFM32 items and daily activities from the perspective of individuals with Type 2 and Type 3 (non-ambulant and ambulant) SMA and their caregivers.

## Methods

### Study overview

**Fig. 1:**
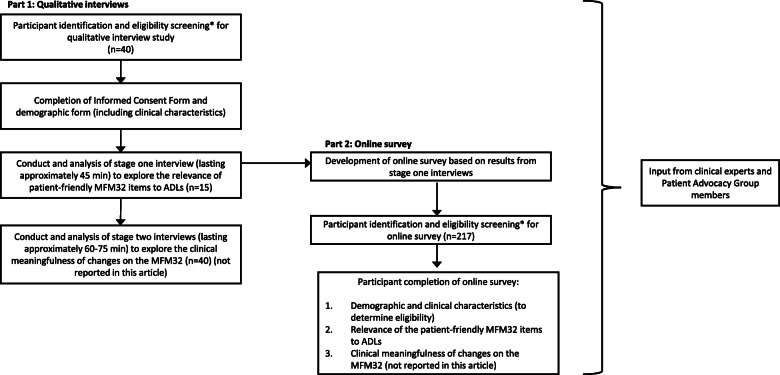
Overview of qualitative interview study and quantitative online survey design. *Notable exclusion criteria included individuals with SMA who were enrolled in a clinical trial or currently being or having previously been treated with risdiplam (EVRYSDI™) or onasemnogene abeparvovec-xioi (ZOLGENSMA®) treatment. However, individuals who were in the maintenance dosing phase of nusinersen (SPINRAZA®) treatment were considered eligible as it was considered to be a sufficient time on treatment for an individual to understand their changes in function and would not influence their perspective of how the patient-friendly MFM32 items relate to ADLs. Individuals who had received nusinersen treatment but discontinued were also included within the quantitative online survey sample. ADL, activities of daily living; MFM32, 32-item motor function measure

This study consisted of two parts as shown in Fig. 1. Part 1 was comprised of in-depth, semi-structured, qualitative telephone interviews involving individuals with SMA and caregivers from the US, between November 2019 and January 2020. Part 2 consisted of an online survey completed by a larger sample of individuals with SMA and caregivers from Canada, France, Germany, Italy, Poland, Spain, the US and the UK, between February 2020 and April 2020. Ethical approval and oversight of the qualitative interview study was provided by Copernicus Group Independent Review Board (IRB), a centralized IRB in the US (approval reference number IRB 20192527). The online survey content included a reduced set of questions from the qualitative interviews. The online survey was conducted following principles consistent with British Healthcare Business Intelligence Association guidelines for Adverse Event reporting and General Data Protection Regulation guidelines, which are applicable to all countries. Participants in both parts of the study were individuals or caregivers of individuals with a self-reported genetic diagnosis of either Type 2, non-ambulant (unable to walk unaided [without braces, assistive devices or person/hand-held assistance] for 10 m or more)  or ambulant (can walk at least 10 m unaided) Type 3 SMA.

### Overview of interview resources relating to the MFM32

The MFM32 measure is comprised of 32 items that assess a range of different motor functional abilities across three functional domains: standing and transfers (D1: 13 items), axial and proximal motor function (D2: 12 items) and distal motor function (D3: 7 items) [10]. These domains assess a broad spectrum of abilities including gross and distal motor functioning of the upper and lower limbs. Each MFM32 item is scored on a 4-point Likert scale from 0 (cannot initiate the task) to 3 (performs the task fully). Item scores are summed, and the raw score is transformed to an overall total score ranging from 0 (severe functional impairment) to 100 (no functional impairment). As part of both the qualitative interview study and the quantitative online survey, eligible participants completed a patient-friendly version of the MFM32 to obtain insights into the perceived level of physical function of the individual with SMA. This patient-friendly version of the MFM32 was developed based on the ‘task to perform’ terminology in the MFM User Manual [18] input from clinical experts and patient advocacy groups. The items of the validated clinician-reported version of the MFM32 were reworded into patient-friendly language by reducing clinical terminology, while maintaining focus on the specific ability assessed by each item in the clinician-reported version. The purpose of the patient-friendly MFM32 was to facilitate participant understanding of the motor function abilities associated with each item of the clinical scale to allow discussion with participants about their perception of how the abilities assessed by the MFM32 items could be related to ADLs.

Since the MFM32 items were not administered to participants using the exact clinical language used in the validated clinician-reported version but instead were modified to ensure patient understanding, they are referred to throughout this manuscript as ‘patient-friendly MFM32’ items. For example:

**Item 14 - validated clinician-reported MFM32 item:** Seated on the chair or in the wheelchair, head in flexion: from head in complete flexion, raises the head then maintains it raised for 5 s, the head stays in midline position throughout the movement and holding position.

**Item 14 - patient-friendly MFM32 item:** When seated and looking at the floor, can you lift your head up and keep it lifted for 5 s?

The patient-friendly version of the MFM32 items were also reordered, based on prior Rasch measurement theory analyses, by increasing level of difficulty to allow exploration of the items most relevant to the individual’s current level of functioning. For the qualitative interviews, the 4-point Likert scale (0–3) of the MFM32 was retained and response options were written in patient-friendly language. Further, the maximal ability was assessed for each item, and intermediate abilities associated with specific scores were not captured as in the validated version of the MFM32. Patient-friendly response options for the qualitative interviews were as follows:

**0:** Unable to start the ability.

**1:** Able to start the ability but not able to finish it.

**2:** Performs the ability but with some help, slowly, without complete control or cannot hold it for long.

**3:** Performs the ability fully.

For the quantitative online survey, the response options were collapsed to “can do (either partially or fully)” and “cannot do”, given that it would not be possible to provide additional clarification to participants or to confirm understanding of more granular response options, if required. Similarly, based on patient advocacy group feedback, some of the patient-friendly MFM32 items were simplified further for the survey in order to be confident that interpretation of the concept would be consistent in the context of the online administration. The patient-friendly MFM32 item and the equivalent clinical item wording are presented in Supplementary Table 1.

### Part 1: qualitative interview study

#### Overview

Participants were identified in collaboration with Cure SMA (a patient advocacy group in the US) and Rare Patient Voice (a non-profit organization that manages patient/caregiver panels) who advertised the study to their SMA communities. Written informed consent to participate was obtained prior to scheduling a telephone interview. Semi-structured telephone interviews were conducted with participants by trained qualitative interviewers in English (the participants’ first language). Interviews aimed to explore the content validity and relevance of the abilities assessed by the patient-friendly MFM32 items to daily activities important to individuals with SMA. Participants enrolled were caregivers of individuals 2 years and older and patients 18 years and older, given the potential complexity of the questions asked in relation to the MFM32 and given the lack of available visualizations of the MFM32 items, which would have been important to facilitate discussion with participants younger than 18 years old. The interview guides used with caregivers of individuals 2 years and older and patients 18 years and older can be viewed in Supplementary File 1 and 2, respectively.

#### Interview procedure

Responses on the patient-friendly version of the MFM32, which was completed prior to the interviews, were used to inform the order of questioning during the interviews. Firstly, for all patient-friendly MFM32 items in which the individual with SMA scored a maximum score of 3 (performs the ability fully), participants were asked: 1) “Tell me about things that you do/the individual with SMA does in your/his/her daily life that involve this movement/ability?” and 2) “Is this an important ability for you/him/her to be able to do? Why/why not?”. Time permitting, participants were also asked these questions in relation to the patient-friendly MFM32 items that the individual with SMA scored a 2, 1 and 0 on.

#### Data analysis

All interviews were audio recorded and transcribed verbatim. The verbatim transcripts were analyzed using thematic analysis methods and ATLAS. Ti software [19]. Thematic analysis is a foundational, theory-free, qualitative analysis method, which offers flexibility to provide a rich, detailed and complex synthesis of data that meets a very specific and applied aim [20, 21]. An induction-abduction approach was taken to identifying themes in the data where themes were identified both by topics emerging directly from the data (inductive inference) and by applying prior knowledge (abductive inference) [22]. This enabled the analysis to remain rooted in the data, allowing participants to identify areas of importance for them, but also to take into consideration prior knowledge. After analyzing each transcript, a list of participant verbatim statements was generated for each coding domain. Concept frequency was determined by counting the number of participants who mentioned a concept (ADL), at least once, during the interview. However, given that not all patient-friendly MFM32 items were discussed with every participant, conceptual saturation analysis could not be performed. Systematic subgroup analysis for SMA type, age and participant respondent (i.e., patient versus caregiver) was not possible due to the small sample size. However, key themes relating to the type of ADLs that were considered relevant were explored and any examples of participants reporting items not being related to ADLs were extracted.

### Part 2: quantitative online survey

#### Overview

Participants eligible to take part in the survey were identified in collaboration with international patient advocacy groups, namely SMA UK, PatientenStimme SMA (Germany), Famiglie SMA (Italy), FundAME (Spain), Fundacja SMA (Poland), AFM Telethon (France) and CureSMA (US and Canada). Patient/caregiver panels were also used to supplement participant identification where necessary. Participants were sent a link to the quantitative online survey which had been translated into their local language and contained an electronic informed consent form where participants completed a tick box indicating consent to participate prior to survey completion. The online survey can be viewed in Supplementary File 3.

#### Survey procedure

As part of the survey, participants were presented in turn with each of the patient-friendly MFM32 items. For each patient-friendly MFM32 item where the response, “can do” was selected, participants were first asked to select any ADLs they considered to be related to the item from a pre-specified list of options. This list of options was developed based on the most frequently reported specific ADLs in the first 10 qualitative interviews. Participants were also presented with an open text field for each patient-friendly MFM32 item in which they could write in any additional ADLs which they considered relevant to the ability not listed in the pre-defined options. Participants also had the option of not selecting an ADL and moving to the next question. Secondly, for the first three patient-friendly MFM32 items where the participants responded “cannot do”, participants were asked to select reasons why some improvement in the ability assessed by the patient-friendly MFM32 item would be considered important. These reasons were from a list of pre-specified options based on the first 10 qualitative interviews. Participants were also presented with an open text field for each patient-friendly MFM32 item in which they could write in any other reasons for importance. However, once a participant selected “cannot do” for five consecutive items, this section of the survey was terminated to avoid asking questions that were not relevant to the individual’s current functional ability.

Participants were also asked to select from a pre-defined list of other symptoms or impacts of SMA that would be important to maintain or improve which were not measured by motor function assessments such as the MFM32. The pre-defined list of options developed by clinical experts and patient advocacy groups were: level of fatigue/lack of energy (tiredness), level of endurance (ability to carry out tasks for longer), voice (tone, pitch, volume), clarity of speech, difficulty sleeping, pain and tremors. Participants were also given the option to specify additional symptoms/impacts by selecting the “other” response option.

#### Data analysis

Survey results were analyzed using PowerBI [23] software whereby concept (ADL) frequency was determined by the number of participants who selected each response option or reported an ADL in the open text field. Due to the number of subgroups by age (5), type (3) and country (8) in the quantitative online survey leading to a wide range in the number of respondents per patient-friendly MFM32 item; therefore, a systematic subgroup comparison was not conducted. Instead, general themes by subgroup were evaluated relating to any obvious differences in the specific ADLs associated with the patient-friendly MFM32 item, and individual item response rates were explored in order to assess the average non-response rate.

## Results

### Sample characteristics

Eight adult individuals with SMA (Type 2 [*n* = 3], non-ambulant Type 3 [*n* = 1], ambulant Type 3 [*n* = 4]) and seven caregivers of individuals with SMA (Type 2 [*n* = 6], ambulant Type 3 [*n* = 1]) from the US took part in the qualitative interview study (Part 1). Of note, no patient-caregiver dyads were recruited, thus providing information relating to 15 different individuals with SMA. In total, 217 participants took part in the quantitative online survey (Part 2), which included 119 individuals with SMA (Type 2 [*n* = 54], non-ambulant Type 3 [*n* = 35], ambulant Type 3 [*n* = 30]) and 98 caregivers of individuals with SMA (Type 2 [*n* = 62], non-ambulant Type 3 [*n* = 16], ambulant Type 3 [*n* = 20]). The participants were from six EU countries, the US and Canada. An overview of the demographic and self-reported clinical characteristics for individuals with SMA and by caregivers on behalf of the individual with SMA are provided in Table 1.

**Table 1:** Demographic/clinical characteristics of individuals with SMA^a^ in qualitative interview study and quantitative online survey

Demographic/clinical characteristics	Qualitative interview (n = 15)	Quantitative online survey (***n*** = 217)
**Total,** n (individual with SMA, caregivers)^b^	15 (8, 7)	217 (119, 98)
**Country,** n (individual with SMA, caregivers)^b^		
US	15 (8, 7)	30 (17, 13)
Canada	0	23 (17, 6)
France	0	28 (15, 13)
UK	0	22 (8, 14)
Germany	0	20 (13, 7)
Italy	0	31 (16, 15)
Spain	0	32 (17, 15)
Poland	0	31 (16, 15)
**Age of individual with SMA**, mean years (min–max)	16 (3–25)	27 (2–59)
**Gender,** n (%)		
Female	11 (73)	134 (62)
Male	4 (27)	83 (38)
**Type of SMA,** n (%)		
Type 2	9 (60)	116 (53)
Type 3 – non-ambulant	1 (7)	51 (24)
Type 3 – ambulant	5 (33)	50 (23)
**Currently receiving/taking treatment to manage SMA,** n (%)		
Yes	13 (87)	102 (47)
No	2 (13)	115 (53)
**Self-reported confirmed diagnosis of scoliosis,** n (%)		
Yes	9 (60)	159 (73)
No	6 (40)	58 (27)
**Self-reported presence of contractures,** n (%)		
Yes	10 (67)	166 (77)
No	5 (33)	45 (21)^c^
**Self-reported daily activities affected,** n (%)		
None of the time	0	N/A
Hardly any of the time	1 (7)	N/A
A little of the time	1 (7)	N/A
Some of the time	1 (7)	N/A
Most of the time	4 (27)	N/A
Almost all of the time	5 (33)	N/A
All of the time	3 (20)	N/A
0–25% of the time	N/A	54 (25)
26–50% of the time	N/A	52 (24)
51–75% of the time	N/A	41 (19)
76–100% of the time	N/A	67 (31)
**Hours per week providing care,** mean (range)	94 (35–168)^b^	79 (0–168)^b^

^a^Please note that the information/data for seven individuals with SMA in the qualitative interview study and 98 individuals with SMA in the quantitative online survey were provided by their caregiver who participated rather than the individual themselves

^**b**^The total sample size and country of participants also includes the caregivers who participated. Hours per week providing care also relates to the caregivers who participated. All other demographic/clinical characteristics relate to the individuals with SMA

^**c**^It is unknown whether the remaining six (3%) individuals with SMA had contractures

*SMA* Spinal muscular atrophy

### Part 1: qualitative interview study results

**Fig. 2:**
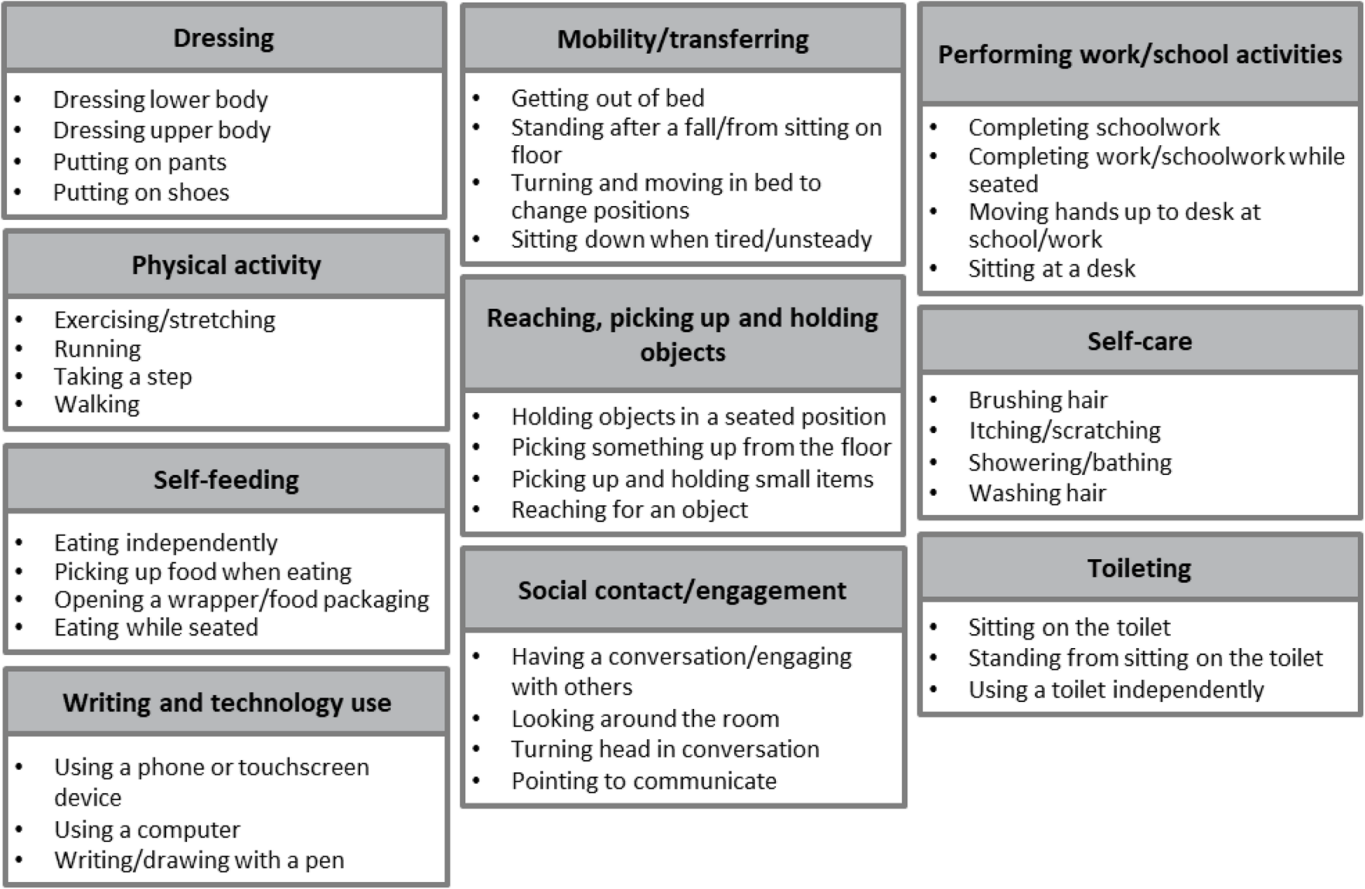
Overview of the 10 key domains and examples of specific ADLs

All 32 patient-friendly MFM32 items were discussed in totality at least once during the 15 interviews. The most frequently reported ADLs considered to be related to the patient-friendly MFM32 items could be grouped into 10 key ADL domains: dressing, mobility/transferring, self-care, self-feeding, reaching, picking up and holding objects, physical activity, writing and technology use, social contact/engagement, toileting and performing work/school activities. Figure 2 demonstrates some of the most frequently reported ADLs for each of the 10 key ADL domains. All patient-friendly MFM32 items were related to one or more ADL domain (range: 2 to 8 related ADL domains per item). In-depth quotes relating to the relevance and content validity of the patient-friendly MFM32 items were obtained (Table 2).

**Table 2:** Sample quotes from qualitative interviews regarding the relevance of the MFM32 items to daily life

MFM32 item	Participant characteristics	Quote
Item 9 (sitting on the floor, maintains position whilst keeping contact between hands; D2)	Caregiver of a 3-year-old individual with Type 2 SMA	“*… you’re* ***worried about her cracking her head or injuring herself*** *in some way … her* ***being able to sit safely allows her more independence*** *to be able to do something on her own and also it,* ***it’s safety regards just her sitting for any kind of daily task****.”*
Item 10 (sitting on the floor, leaning forward to touch a ball; D2)	22-year-old individual with non-ambulant Type 3 SMA	*“So basically****anything that I need to reach****… I work in a research lab, and so I****reach like different … equipment****in the lab and just reaching my computer to do like****daily work****stuff …****feeding myself****.”*
Item 11 (stands up from sitting on the floor; D1)	Caregiver of a 9-year-old individual with Type 2 SMA	*“If he could stand up from seated,****that would help at school****. That would****help at home****. Just****being able to be on the floor to pick something up****, to****play with our dogs****… any of those would be****super helpful****.”*
Item 14 (sitting on the floor, lifts head up and keeps lifted for 5 s; D2)	24-year-old individual with Type 2 SMA	*“that’s like****most of my activities****… so doing anything … I have to obviously have my head up, so … when I’m****sitting up****,****which is 90% of the day****… so like when I’m****eating****… if I’m****working****… if I’m just****talking****to someone …****reading a book****…****using my phone****…****my head is constantly in that position****.”*
Item 15 (bringing arms up to place both hands on top of the head; D2)	24-year-old individual with Type 2 SMA	*“That would just be helpful for doing any … motions around your face …****wiping hair out of your face****or maybe****putting on makeup****or something like that …****washing your hair****… So,****there’s a lot of daily tasks that use that activity and that motion****.”*
Item 16 (sitting at a table, reaches forward to touch a pencil in front; D2)	18-year-old individual with ambulant Type 3 SMA	*“So I use this one … when I’m****at work****and just like in class in general or eating … like****grabbing a fork or grabbing my pencil****to write or even just****typing on the keyboard****… So I feel like****this one is a really important one****to have … it****comes up a lot****...I feel like that’s like****important for everyone****as a whole.”*
Item 17 (picking up 10 coins and holding in hand for 20 s; D3)	Caregiver of a 6-year-old individual with Type 2 SMA	*“So being****able to write****… to****hold an object****… for an extended period of time … being able****to eat****… I keep going back to, you know, the****items that are so important too with everyday living****… he loves to****play games****… So, you know, to hold little characters or whatever and play with, play with those as well.”*
Item 21 (picking up a ball in front and turn hand over; D3)	Caregiver of a 3-year-old individual with Type 2 SMA	*“… it’s allowing you****to take care of those****kind of daily what seem like****mundane tasks****, but those****daily tasks****that she wouldn’t otherwise be able to do … working on that, that turning mechanism for****opening a door****or****opening a drawer****…****turning and opening things.****”*
Item 23 (placing forearms and/or hands on table; D2)	18-year-old individual with ambulant Type 3 SMA	*“So this one I would say****I use a lot****…****on a day to day****… especially like when I’m****eating****… putting my****hands up on the table****… or when I’m****at work****and I’m working on something … just****moving my hands up to my desk****as well.”*

MFM32, 32-item Motor Function Measure

Table 3 presents each of the key ADL domains, and the specific ADL within each domain that was most frequently reported for each of the items assessed in the MFM32. For example, putting on shoes (key ADL domain: dressing) was the most frequently reported specific ADL for item 4 (pulling up the foot). In the interviews, there were subtle differences in the types of ADLs raised as related to patient-friendly MFM32 items by individuals with Type 2 SMA compared with those with Type 3 SMA and types of ADLs raised by individuals with SMA compared with caregivers. However, there were no obvious differences between any of the items in terms of the ability of participants to identify an ADL as related to each MFM32 item.

**Table 3:** Most frequently reported ADLs in relation to MFM32 item

ADL domain	MFM32 item	Most frequently reported daily activity	Reported in interviews	Reported in survey
**Dressing**	Item 4 (Pulling up the foot)^A, B^	Putting on shoes	✓	✓
	Item 3 (Bringing knee to chest)^A, B^	Dressing lower body	✓	✓
	Item 6 (Raise pelvis)^A, B^	Putting on pants	✓	✓
	Item 5 (Bringing hand to opposite shoulder)^A^	Dressing upper body	✓	✓
	Item 26 (Standing on one foot)^A^	Dressing lower body	✓	✓
**Mobility/transferring**	Item 7 (Roll from lying on front to back)^A, B^	Turning and moving in bed to change positions	✓	✓
	Item 8 (Lying down to sitting up)^A, B^	Getting out of bed	✓	✓
	Item 11 (Sit to stand)^A, B^	Stand after a fall/from sitting on floor	✓	✓
	Item 1 (Turning head)^A^	Adjusting position in bed	✓	✓
	Item 2 (Lifting head)^A^	Lifting head to move pillow/getting out of bed	✓	✓
	Item 25 (Stand without support)^A^	Stand from sitting	✓	●
	Item 29 (Walking on a line)^A^	Walking around the house	✓	✓
	Item 12 (Sitting down on a chair from standing) ^A^	Sitting down when tired/unsteady	✓	●
	Item 24 (Standing up from sitting on chair) ^A^	Stand up from sitting at dinner table/to change position/when carrying objects	✓	✓
**Self-care**	Item 15 (Bring arms up to place both hands on top of the head)^A, B^	Brushing hair	✓	✓
	Item 5 (Bringing hand to opposite shoulder)^B^	Itching/scratching	✓	✓
**Self-feeding**	Item 23 (Place forearms and/or hands on table)^A, B^	Eating independently	✓	✓
	Item 21 (Turning a ball over in hand)^A, B^	Picking up food when eating	✓	✓
	Item 16 (Extending elbow to touch a pencil)^A, B^	Picking food off a table without help	✓	✓
	Item 20 (Tearing a sheet of paper)^A^	Opening a wrapper/food packaging	✓	✓
	Item 13 (Maintain a seated position)^B^	Eating while seated	✓	✓
**Reaching, picking up and holding objects**	Item 17 (Picking up coins)^A, B^	Picking up and holding small items	✓	✓
	Item 9 (Sitting on the floor)^A, B^	Holding objects in a seated position	✓	✓
	Item 10 (Leaning towards a ball)^A, B^	Reaching an object	✓	✓
	Item 27 (Touching the floor while standing)^A, B^	Touching the floor to pick up something	✓	✓
	Item 32 (Squatting)^A, B^	Picking something up from the floor	✓	✓
**Physical activity**	Item 28 (Walking on heels)^A, B^	Walking	✓	✓
	Item 30 (Running)^A, B^	Exercising	✓	✓
	Item 31 (Hopping)^A, B^	Exercising/playing sport/hopping	✓	✓
	Item 29 (Walking on a line)^B^	Walking	✓	✓
	Item 26 (Standing on one foot)^B^	Taking a step/walking	✓	✓
**Writing and technology use**	Item 22 (Pointing at drawings)^A, B^	Using a phone or other device/touchscreen device	✓	✓
	Item 18 (Going around the edge of a CD)^A, B^	Using a touchscreen device	✓	✓
	Item 19 (Pick up pencil and draw loops)^A, B^	Writing/drawing with a pen	✓	✓
	Item 20 (Tearing a sheet of paper)^B^	Using your hands to tear a piece of paper	✓	✓
**Social contact/engagement**	Item 14 (Raise the head from the chest)^A, B^	Having a conversation/engaging with others	✓	✓
	Item 1 (Turning head)^A, B^	Looking around the room	✓	✓
	Item 2 (Lifting head)^B^	Looking around the room	✓	✓
**Toileting**	Item 25 (Stand without support)^B^	Using a toilet independently	✓	✓
	Item 12 (Sitting down on a chair from standing)^B^	Using a toilet independently	✓	✓
	Item 24 (Standing up from sitting on chair)^B^	Standing from sitting on toilet	✓	✓
**Performing work/school activities**	Item 13 (Maintain a seated position)^A^	Doing work/schoolwork while seated	✓	✓

^A^ The item was most frequently associated with the ADL based on the qualitative interview data

^B^ The item was most frequently associated with the ADL based on the survey data

✓ The specific aspect of the ADL was reported in relation to the patient-friendly MFM32 item in the qualitative interviews and/or quantitative online survey

● The specific aspect of the ADL was not included as a response option in the quantitative online survey and was also not reported in the free-text response option by any respondents

*ADL* Activities of daily living; MFM32, 32-item Motor Function Measure

Participants were also asked to briefly comment on the importance of each patient-friendly MFM32 item. For all of the patient-friendly MFM32 items, at least one or more participant was able to state the relevance to daily life. A sample of quotes regarding the relevance of the MFM32 concepts items to daily life is presented in Table 2. The two most frequently reported reasons as to why the items were considered important included “the movement/skill is helpful in daily life” (131 mentions across the 15 interviews) and “the movement/skill allows for increased independence” (129 mentions across the 15 interviews).

Of the 15 participants interviewed, the number of patient-friendly MFM32 items discussed per interview ranged from 22 to 32. Of note, there were five participants who indicated that a patient-friendly MFM32 item (range 1–3 items per participant, relating to seven items in total) was not related to an everyday activity in their life. While one of the seven items (tearing a sheet of paper) was raised by two participants as not related to an ADL, the other six items were raised by one participant, indicating individual variability rather than a consistent trend towards a specific patient-friendly MFM32 item not being relevant to daily life.

### Part 2: quantitative online survey results

Responses from the quantitative online survey confirmed that all patient-friendly MFM32 items were considered related to ADLs by the majority of participants (see Supplementary Table 1). Table 3 shows the specific ADL for each domain most frequently reported for the patient-friendly MFM32 items. The ADL most frequently associated with each patient-friendly MFM32 item according to the quantitative online survey could be grouped into the same ADL domains as those defined in the qualitative interview results. Nine patient-friendly MFM32 items (Items 2, 5, 12, 13, 20, 24, 25, 26, 29, defined in Table 3) were most frequently associated with a different ADL domain in the quantitative online survey results compared to the qualitative interviews. One patient-friendly MFM32 item (Item 1 defined in Table 3) was more frequently associated with an additional ADL domain in the qualitative interviews but not in the quantitative online survey. In both circumstances, items are included twice in the table to demonstrate this. An average of 9% (range 0–20%) of participants selected the “other” response option per patient-friendly MFM32 item; however, an alternative ADL was not always provided. In addition, on average 12% (range 0–24%) of participants did not select any ADL per item. Further details on the response frequencies for each item can be found in Supplementary Table 1. Generally, any differences in the specific ADLs associated with the patient-friendly MFM32 items appeared to be driven by SMA type or age group of the individual rather than the country. For example, for individuals aged 2–5 years, the ADL most frequently considered to be related to the patient-friendly version of item 22 (pointing at drawings) was “reaching for objects”, whereas for other age groups the ADL most frequently considered to be related to item 22 was “using electronic devices”. For individuals with ambulant Type 3 SMA, the ADL most frequently considered to be related to the patient-friendly version of item 13 (maintain a seated position) was “maintaining seated position without backrest”, whereas for individuals with Type 2 and non-ambulant Type 3 SMA the ADL most frequently considered to be related to item 13 was “sitting in a chair/wheelchair”. Nevertheless, for the patient-friendly MFM32 items participants indicated they “can do”, the vast majority selected one or more specific ADL per patient-friendly MFM32 item, demonstrating the measure was relevant to the daily lives of both individuals with Type 2 or (non-ambulant and ambulant) Type 3 SMA across age groups (ranging from 2 to 59 years old).

**Fig. 3:**
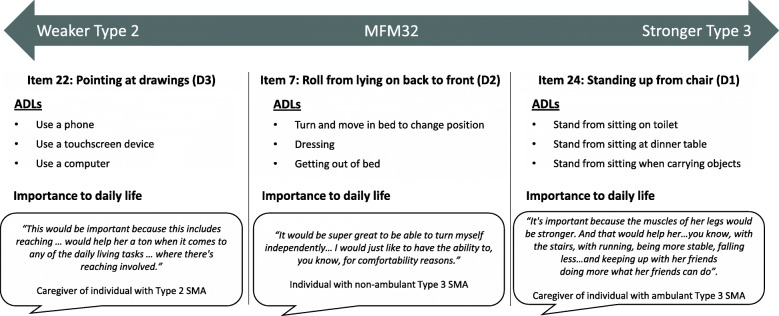
Example MFM32 items, associated ADLs and importance to daily life across the functional spectrum of the MFM32

Figure 3 shows the relevance of the MFM32 items across the functional spectrum of Types 2 and 3 SMA. There were no major differences in the response rate (proportion of respondents that selected at least one ADL or used the “other” response options versus the proportion of respondents that did not select an ADL and did not use the ‘other’ response option [non-response]) by SMA type or age group. However, there were subtle differences in the response rate by country, with a higher average response rate across the 32 items in the US (94%), Canada (92%) and Germany (94%) compared with France (83%), the UK (87%), Italy (87%), Poland (86%), and Spain (80%). Of the first three patient-friendly MFM32 items that participants indicated that they “cannot do”, participants were also asked to report the reasons why gaining some ability on each of these would be important. Again, among the most frequently reported reasons were that the gain in ability would support increased independence (366 reports across items/respondents) and the ability is a helpful skill in daily life (293 reports across items/respondents).

**Fig. 4:**
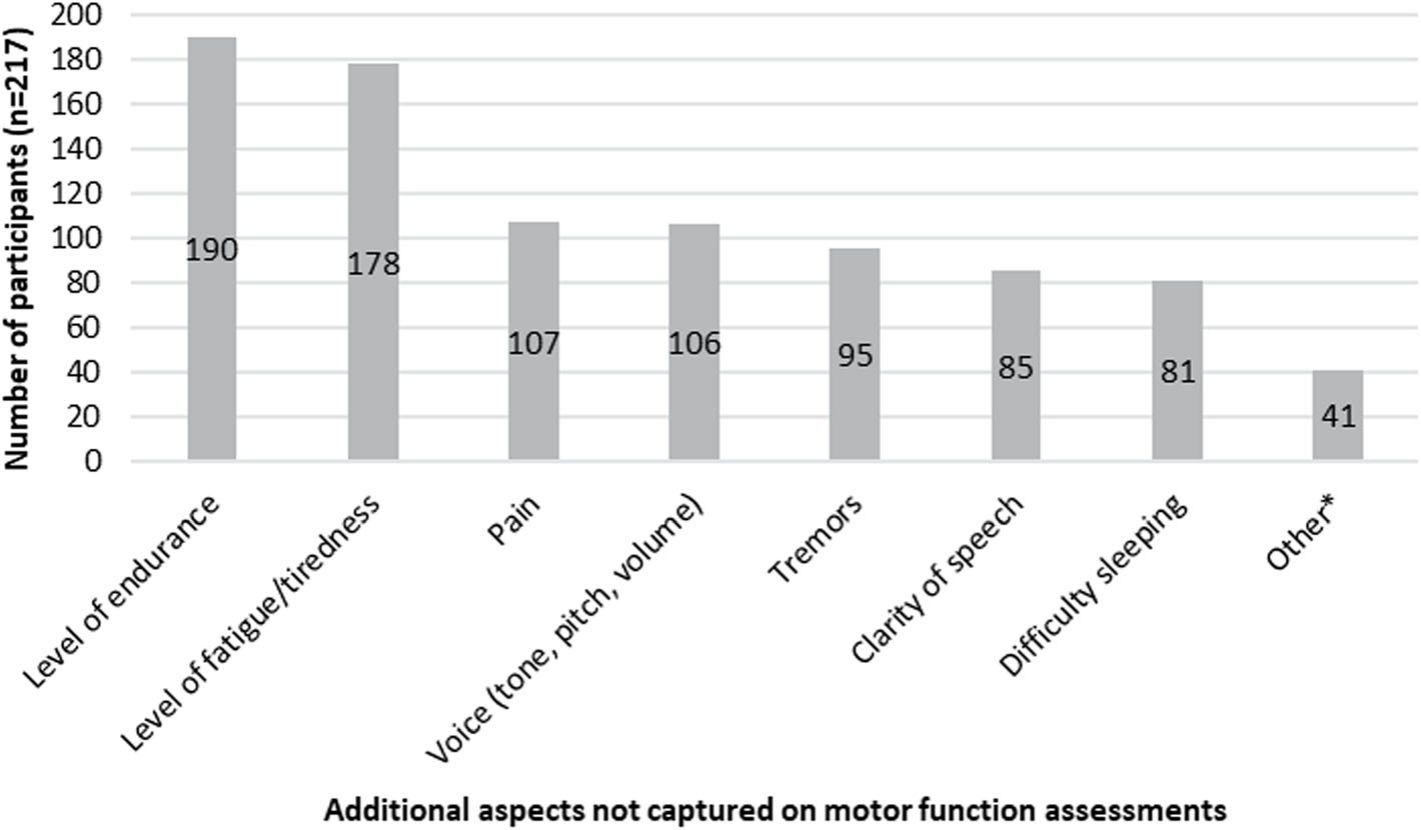
Additional symptoms and impacts that are important to maintain/improve not captured on motor function assessments. *Breathing/respiratory function (*n *= 12), muscle strength (*n *= 7), chewing/coughing/swallowing (*n *= 4), mental/psychological problems (*n* = 4), elimination of contractures/scoliosis (*n* = 3), general physical safety (*n* = 2), ability to feed oneself/brush teeth/sign name (*n* = 1), comfort (*n* = 1), exercise assessment (*n* = 1), losing weight (*n* = 1), moving position (*n* = 1), poor blood circulation/cold feet (*n* = 1), sexual life (*n* = 1), social interactions (*n* = 1), reason unclear (*n* = 1)

The most frequently reported additional symptoms and impacts that were considered important to measure in addition to motor function were levels of endurance (*n* = 190) and levels of fatigue/lack of energy (*n* = 178; Fig. 4). This observation was consistent across SMA type, age and country. There were *n* = 41 participants that selected the “other” response option; however, additional symptoms/impacts were not always provided on selection of this option. Where a symptom/impact was provided, these were analyzed and grouped into themes listed as a footnote of Fig. 4.

## Discussion

This two-part study demonstrates how functional abilities assessed by the MFM32 relate to important ADLs for individuals with Type 2 SMA and non-ambulant and ambulant Type 3 SMA. In doing so, the study provided evidence supporting the relevance of the functional abilities of the MFM32 to ADLs from a patient and caregiver perspective, an important step in the validation process. Establishing relevance is strongly advocated by regulators and HTA bodies, and provides support for the use of the MFM32 in a clinical trial setting [15–17]. A previous study by Pera et al. in 2017, demonstrated the clinical relevance of the HFMSE items according to patients [11]. In addition, the original Upper Limb Module (ULM) was developed in collaboration with clinicians, physical therapists, researchers, and patient advocacy groups and measures motor function performance through items that relate to meaningful ADLs [12, 24]. Together, these studies demonstrate how the patient perspective has been vital to determining the clinical relevance of these scales, which is an important consideration for the context of clinical trials.

After 15 qualitative interviews with both individuals and caregivers of individuals with SMA were conducted, 10 key ADL domains were identified as being related to the patient-friendly MFM32 items: dressing, mobility/transferring, self-care, self-feeding, reaching, picking up and holding objects, physical activity, writing and technology use, social contact/engagement, toileting and performing work/school activities. Previous research has shown that the majority of these ADL domains have a major impact on quality of life for individuals with Type 2 and Type 3 SMA [7]. Therefore, these results demonstrate that the patient-friendly MFM32 items are related to important daily activities across the disease spectrum of Type 2 and Type 3 SMA (non-ambulant and ambulant), as well as across age groups (3–59 years old).

The two-part design of this study was a strength of this research. In-depth qualitative data was substantiated by the findings of the quantitative online survey which represented 217 participants across eight countries. Each specific aspect of an ADL domain that was included as a response option in the quantitative online survey (based on the qualitative interview findings) was endorsed by multiple participants per item, demonstrating a high degree of consistency between the findings of the qualitative interviews conducted in a US sample and of the quantitative online survey conducted in eight countries. When considered together, the results from both parts of the study provide evidence that the specific ADLs reported for each patient-friendly MFM32 item could be considered representative of ADLs important to the wider Types 2 and 3 SMA population.

It is difficult to interpret and draw conclusions regarding subgroup differences in the specific ADL discussed/selected in relation to each patient-friendly MFM32 item, due to the small sample size in the qualitative interviews (*n* = 15) and the wide range in number of respondents per patient-friendly MFM32 item by subgroup in the quantitative online survey. In some instances, the relevance of the specific ADL in the pre-defined list varied slightly depending on the age of the individual (e.g., reaching for objects being most relevant for 2–5-year-olds and using an electronic device being most relevant for all other ages) or the type of SMA and residual functional ability of the individual (e.g., maintaining a seated position with no backrest being most relevant for ambulant individuals with Type 3 SMA vs sitting in a chair or a wheelchair for individuals with Type 2 or non-ambulant Type 3 SMA). This was to be expected due to the heterogeneity of symptom presentation in SMA. However, despite the range of functional abilities in the sample, the patient-friendly MFM32 items were shown to assess abilities relevant to the daily activities of all individuals, regardless of their SMA type or age. Thus, these findings can be attributed to the broad spectrum of abilities assessed by the three domains of the measure, covering both the gross and distal motor functioning of the upper and lower limbs. Given the wide range of abilities assessed by the measure, the MFM32 could be useful to implement in clinical trials of a long duration during which patients may undergo changes in their functional status [25].

Additionally, although the qualitative interviews were conducted with participants from the US only, the quantitative online survey obtained input from individuals and caregivers from seven different countries in addition to the US. The findings of the survey and interviews were consistent, providing confidence that the themes that arose from the qualitative interviews can be generalized to other countries/cultures. Further, relatively few additional ADLs were reported using the “other” free-text response option in the quantitative online survey, providing evidence that the ADLs identified in the Part 1 interviews were adequately comprehensive. In addition, in the quantitative online survey the proportion of non-responses was relatively low suggesting that participants were generally able to select an ADL from the pre-defined list or provide another suggestion. Participants were not asked as to why they did not select an ADL or provide another suggestion (non-response) and therefore it is not possible to make conclusions as to why this might have occurred. However, item non-response is relatively common in online surveys [26] and the longer length of this online survey may have influenced the non-response rates observed [27].

A further strength of this research was the adaptive nature of the qualitative interviews and quantitative online surveys. Both parts of the study were tailored to the current motor functioning level of the individual with SMA based on the responses provided on the patient-friendly version of the MFM32. Discussions in the qualitative interviews were focused on the patient-friendly items that the individual with SMA could fully complete (a score of 3). Patient-friendly items in which the individual scored a 2, 1 and 0 were only explored if time permitted. In the quantitative online survey, participants were only asked to report specific ADLs that could be considered relevant to items where the response, “can do” was selected. Although this relies on the accuracy of reporting, it reduces the burden of response and minimizes any potential distress caused by asking about abilities which the participant may no longer have. However, insights on whether losing these abilities were important may not have been captured and limits this approach. Further, when completing the quantitative online survey, participants were asked to select ADLs from a predefined list. While a free-text option was provided for participants to indicate ADLs that were not listed, the presence of the predefined list may have biased the thinking towards the ADLs listed.

A limitation of this study was that it was not possible to use the clinician-reported version of the MFM32 to facilitate discussion as the clinical language of the MFM32 manual and associated items was deemed inappropriate for a self-reported assessment by patients and caregivers. A patient-friendly version of the MFM32 was created to support discussion on how abilities assessed by the clinical MFM32 item could be related to specific ADLs. The patient-friendly version of the MFM32 focused on describing the functional ability associated with achieving a maximum score of 3, as opposed to describing compensations of movement or intermediate functions associated with a score of 1 or 2. For example, for the patient-friendly version of the MFM32 item 15 the ability presented was “bringing two hands to the head”, which is associated with the maximum score of 3 on the MFM32, while a score of 2 on this item indicates the ability to bring the hands to the mouth which was not discussed in the patient-friendly version. Further, as this assessment was completed by the individual with SMA or their caregiver, it was not possible to obtain a clinician-confirmed MFM32 score which could have provided a more accurate assessment of functional ability. In addition, since the MFM32 only assesses motor function and no other aspects of SMA, the conceptual association between an assessment of motor function and ADLs was the focus of this research. Other measures are needed to explore the importance of the additional clinical features described in Fig. 4 and to ensure a holistic measurement of the SMA symptoms and impacts of importance to patients and their families.

A further limitation was the lack of conceptual saturation analysis in the qualitative interview study, given the small numbers of participants in each subgroup. However, as the intent was to understand the relationship between the patient-friendly MFM32 items and everyday activities and not to develop a new patient-reported outcome measure, it was not deemed essential to conduct a conceptual saturation exercise as would be required for more traditional concept elicitation interviews and associated instrument development. In addition, it can be assumed that the ADL domains described are comprehensive, due to the infrequent use of the “other” response options and are unlikely to vary substantially between the subgroups based on the consistency of the results. Further, the patient-friendly MFM32 items were shown to capture several important daily activities for individuals with Type 2 and Type 3 SMA, even though conceptual saturation was not assessed in the study.

It is important to note that a relationship between the patient-friendly MFM32 item and an everyday activity does not mean that change on an item would necessarily lead to changes in that specific daily activity as there are other factors that would need to be considered such as clinical (e.g., degree of scoliosis or contractures) and situational characteristics (e.g., the size and positioning of the object and use of assistive devices). As such, future research could seek to investigate the correlation between the MFM32 and a scale such as the SMA Independence Scale (SMAIS), a patient and caregiver-reported measure of the level of assistance required from another individual to perform ADLs. This could provide an additional objective assessment of the relationship between motor function on the MFM32 and the ability to perform ADLs. Interviewing younger children and adolescents to understand how they consider MFM32 items to be related to ADLs is also a potential opportunity for future research, using alternative methodologies (e.g., use of videos to explain the MFM32) to aid understanding of the functional abilities being assessed in this younger age range.

## Conclusions

In conclusion, this study provides additional, in-depth evidence to support the relevance of the functional abilities assessed by the MFM32 to individuals with Type 2 and Type 3 SMA. Specifically, the patient-friendly MFM32 items were shown to relate to 10 key ADL domains that are important and relevant to both individuals with Type 2 and Type 3 (non-ambulant and ambulant) SMA. Even though the MFM32 was originally designed for individuals of all neuromuscular diseases, these findings provide supportive evidence demonstrating that the patient-friendly MFM32 items are relevant to ADLs from the perspective of individuals with Types 2 and 3 SMA and their caregivers, and abilities important to everyday life. Further research investigating what constitutes a clinically meaningful change on the MFM32 from a patient/caregiver perspective through anchor-based and distribution-based analyses of thresholds of clinically meaningful change is warranted.

## Supplementary Information

**Additional file 1: Table S1.** Detailed analysis of responses to the patient-friendly MFM32 items in quantitative survey.

**Additional file 2.** Supplementary File 1. Qualitative interview guide for caregivers.

**Additional file 3.** Supplementary File 2. Qualitative interview guide for patients.

**Additional file 4.** Supplementary File 3. Online patient and caregiver survey.

## Data Availability

Data generated from this study are not publicly available; additional data may be provided by the authors upon reasonable request.

## Abbreviations

ADL: Activities of daily living
COA: Clinical Outcome Assessment
HFMSE: Hammersmith Functional Motor Scale Expanded
HTA: Health Technology Assessment
IRB: Independent Review Board
MFM32: 32-item Motor Function Measure
RULM: Revised Upper Limb Module
ULM: Upper Limb Module
SMA: Spinal muscular atrophy
*SMN1*: Survival of motor neuron gene

## References

[1] Lunn MR, Wang CH (2008). Spinal muscular atrophy. Lancet (London, England).

[2] Wang CH, Finkel RS, Bertini ES, Schroth M, Simonds A, Wong B, Aloysius A, Morrison L, Main M, Crawford TO, Trela A, Participants of the International Conference on SMA Standard of Care (2007). Consensus statement for standard of care in spinal muscular atrophy. J Child Neurol.

[3] Chen TH (2020). New and Developing Therapies in Spinal Muscular Atrophy: From Genotype to Phenotype to Treatment and Where Do We Stand?. Int J Mol Sci.

[4] Kaufmann P, McDermott MP, Darras BT, Finkel R, Kang P, Oskoui M (2011). Observational study of spinal muscular atrophy type 2 and 3: functional outcomes over 1 year. Arch Neurol.

[5] Mercuri E, Finkel RS, Muntoni F, Wirth B, Montes J, Main M, Mazzone ES, Vitale M, Snyder B, Quijano-Roy S, Bertini E, Davis RH, Meyer OH, Simonds AK, Schroth MK, Graham RJ, Kirschner J, Iannaccone ST, Crawford TO, Woods S, Qian Y, Sejersen T, Muntoni F, Wirth B, Tiziano FD, Kirschner J, Tizzano E, Topaloglu H, Swoboda K, Laing N, Kayoko S, Prior T, Chung WK, Wu SM, Montes J, Mazzone E, Main M, Coleman C, Gee R, Glanzman A, Kroksmark AK, Krosschell K, Nelson L, Rose K, Stępień A, Vuillerot C, Vitale M, Snyder B, Quijano-Roy S, Dubousset J, Farrington D, Flynn J, Halanski M, Hasler C, Miladi L, Reilly C, Roye B, Sponseller P, Yazici M, Hurst R, Bertini E, Tarrant S, Barja S, Bertoli S, Crawford T, Foust K, Kyle B, Rodan L, Roper H, Seffrood E, Swoboda K, Szlagatys-Sidorkiewicz A (2018). Diagnosis and management of spinal muscular atrophy: part 1: recommendations for diagnosis, rehabilitation, orthopedic and nutritional care. Neuromuscul Disord.

[6] Ryan MM, De Vivo DC, Bertini E, Hwu WL, Crawford TO, Swoboda KJ (2019). Nusinersen in infants who initiate treatment in a presymptomatic stage of spinal muscular atrophy (SMA): interim results from the phase 2 nurture study. J Neurol Sci.

[7] Rouault F, Christie-Brown V, Broekgaarden R, Gusset N, Henderson D, Marczuk P, Schwersenz I, Bellis G, Cottet C (2017). Disease impact on general well-being and therapeutic expectations of European type II and type III spinal muscular atrophy patients. Neuromuscul Disord.

[8] Cruz R, Lenz M, Belter L, Hobby K, Jarecki J, Smart T (2018). CureSMA: Voice of the patient report.

[9] Qian Y, McGraw S, Henne J, Jarecki J, Hobby K, Yeh WS (2015). Understanding the experiences and needs of individuals with spinal muscular atrophy and their parents: a qualitative study. BMC Neurol.

[10] Berard C, Payan C, Hodgkinson I, Fermanian J, Group MFMCS (2005). A motor function measure for neuromuscular diseases. Construction and validation study. Neuromuscul Disord.

[11] Pera MC, Coratti G, Forcina N, Mazzone ES, Scoto M, Montes J (2017). Content validity and clinical meaningfulness of the HFMSE in spinal muscular atrophy. BMC Neurol.

[12] Mazzone E, Bianco F, Martinelli D, Glanzman AM, Messina S, De Sanctis R (2011). Assessing upper limb function in nonambulant SMA patients: development of a new module. Neuromuscul Disord.

[13] Vuillerot C, Payan C, Iwaz J, Ecochard R, Berard C, Group MFMS (2013). Responsiveness of the motor function measure in patients with spinal muscular atrophy. Arch Phys Med Rehabil.

[14] Trundell D, Le Scouiller S, Gorni K, Seabrook T, Vuillerot C, the SMSG (2020). Validity and Reliability of the 32-Item Motor Function Measure in 2- to 5-Year-Olds with Neuromuscular Disorders and 2- to 25-Year-Olds with Spinal Muscular Atrophy. Neurol Ther.

[15] Food and Drug Administration (2018). Patient-Focused Drug Development Guidance: Methods to Identify What is Important to Patients and Select, Develop or Modify Fit-for-Purpose Clinical Outcome Assessments. Discussion document.

[16] Food and Drug Administration (2019). Patient-Focused Drug Development: Methods to Identify What Is Important to Patients, Guidance for Industry, Food and Drug Administration Staff, and Other Stakeholders. Draft guidance.

[17] Committee for Medicinal Products for Human Use (2005). Reflection paper on the regulatory guidance for the use of health-related quality of life (HRQL) measures in the evaluation of medicinal products.

[18] Bérard C, Girardot F, Payan C. User’s Manual: MFM-32 & MFM-20. https://mfm-nmd.org/get-a-user-manual/?lang=en Accessed January 2021.

[19] ATLAS.ti (2013). Scientific Software Development GmbH B, Germany. Atlas.ti.software version 7.

[20] Braun V, Clarke V (2006). Using thematic analysis in psychology. Qual Res Psychol.

[21] Kerr C, Nixon A, Wild D (2010). Assessing and demonstrating data saturation in qualitative inquiry supporting patient-reported outcomes research. Expert Rev Pharmacoecon Outcomes Res.

[22] Kelle U (2005). “Emergence” vs. “forcing” of empirical data? A crucial problem of “Grounded Theory” reconsidered. Forum Qual Soc Res.

[23] Data Visualization | Microsoft Power BI. https://powerbi.microsoft.com/en-us/ (2020). Accessed.

[24] Mazzone ES, Mayhew A, Montes J, Ramsey D, Fanelli L, Young SD, Salazar R, de Sanctis R, Pasternak A, Glanzman A, Coratti G, Civitello M, Forcina N, Gee R, Duong T, Pane M, Scoto M, Pera MC, Messina S, Tennekoon G, Day JW, Darras BT, Vivo DC, Finkel R, Muntoni F, Mercuri E (2017). Revised upper limb module for spinal muscular atrophy: development of a new module. Muscle Nerve.

[25] Mercuri E, Mayhew A, Muntoni F, Messina S, Straub V, Van Ommen GJ (2008). Towards harmonisation of outcome measures for DMD and SMA within TREAT-NMD; report of three expert workshops: TREAT-NMD/ENMC workshop on outcome measures, 12th--13th May 2007, Naarden, The Netherlands; TREAT-NMD workshop on outcome measures in experimental trials for DMD, 30th June--1st July 2007, Naarden, The Netherlands; conjoint Institute of Myology TREAT-NMD meeting on physical activity monitoring in neuromuscular disorders, 11th July 2007, Paris, France. Neuromuscul Disord.

[26] Scott A, Jeon S-H, Joyce CM, Humphreys JS, Kalb G, Witt J (2011). A randomised trial and economic evaluation of the effect of response mode on response rate, response bias, and item non-response in a survey of doctors. BMC Med Res Methodol.

[27] Sahlqvist S, Song Y, Bull F, Adams E, Preston J, Ogilvie D (2011). Effect of questionnaire length, personalisation and reminder type on response rate to a complex postal survey: randomised controlled trial. BMC Med Res Methodol.

