# Correction to: Understanding the relationship between the 32-item motor function measure and daily activities from an individual with spinal muscular atrophy and their caregivers’ perspective: a two-part study

**DOI:** 10.1186/s12883-021-02307-4

**Published:** 2021-09-13

**Authors:** Tina Duong, Jessica Braid, Hannah Staunton, Aurelie Barriere, Fani Petridis, Johannes Reithinger, Rosangel Cruz, Jill Jarecki, Mencia De Lemus, Nicole Gusset, Ria Broekgaarden, Sharan Randhawa, Jessica Flynn, Rob Arbuckle, Sonia Reif, Lida Yang, Angela De Martini, Carole Vuillerot

**Affiliations:** 1 Department of Neurology and Neurological Sciences, Stanford University, Stanford, CA USA; 2 Roche Products Limited, Welwyn Garden City, UK; 3 Department of Pediatric Physical Medicine and Rehabilitation, Hôpital Mère Enfant, CHU-Lyon, Lyon University, Lyon, France; 4 F. Hoffmann-La Roche Ltd, Basel, Switzerland; 5 CureSMA, Elk Grove Village, IL USA; 6 SMA Europe Freiburg, Freiburg, Germany; 7 FundAME, Madrid, Spain; 8 SMA Schweiz, Swiss Patient Organisation for Spinal Muscular Atrophy, Heimberg, Switzerland; 9 SMA Europe and Vereniging Spierziekten Nederland, Baarn, The Netherlands; 10 Adelphi Values, Patient-Centered Outcomes, Adelphi Mill, Bollington, Cheshire UK; 11 Charles River Associates Inc, Zurich, Switzerland; 12 Neuromyogen Institute, CNRS UMR 5310 – INSERM U1217, Université de Lyon, Lyon, France

**Correction to: BMC Neurol 21, 143 (2021)**

**https://doi.org/10.1186/s12883-021-02166-z**

Following publication of the original article [1], the authors reported the following errors:

- Figure 1. Information regarding the population inclusion/exclusion criteria was previously missing and has been added as a footnote.
- Part 2 quantitative online survey results: The text describing the activity domains (ADLs) most frequently raised for each item in the online survey has been corrected to nine items being more frequently associated with a different ADL domain in the quantitative online survey when compared to the interviews. The overall skip rate (12%) and country response percentage rates for the following countries US (94%), Canada (92%), France (83%), UK (87%), Poland (86%), and Spain (80%) have been corrected.
- Table 3. The footnote letters for items 4, 8, 12, 15, 24, 25, 30, and 31 have been corrected.

**Table 3:** Most frequently reported ADLs in relation to MFM32 item

ADL domain	MFM32 item	Most frequently reported daily activity	Reported in interviews	Reported in survey
**Dressing**	Item 4 (Pulling up the foot)^A, B^	Putting on shoes	✓	✓
	Item 3 (Bringing knee to chest)^A,B^	Dressing lower body	✓	✓
	Item 6 (Raise pelvis)^A,B^	Putting on pants	✓	✓
	Item 5 (Bringing hand to opposite shoulder)^A^	Dressing upper body	✓	✓
	Item 26 (Standing on one foot)^A^	Dressing lower body	✓	✓
**Mobility/transferring**	Item 7 (Roll from lying on front to back)^A,B^	Turning and moving in bed to change positions	✓	✓
	Item 8 (Lying down to sitting up)^A,B^	Getting out of bed	✓	✓
	Item 11 (Sit to stand)^A,B^	Stand after a fall/from sitting on floor	✓	✓
	Item 1 (Turning head)^A^	Adjusting position in bed	✓	✓
	Item 2 (Lifting head)^A^	Lifting head to move pillow/getting out of bed	✓	✓
	Item 25 (Stand without support)^A^	Stand from sitting	✓	•
	Item 29 (Walking on a line)^A^	Walking around the house	✓	✓
	Item 12 (Sitting down on a chair from standing) ^A^	Sitting down when tired/unsteady	✓	•
	Item 24 (Standing up from sitting on chair) ^A^	Stand up from sitting at dinner table/to change position/when carrying objects	✓	✓
**Self-care**	Item 15 (Bring arms up to place both hands on top of the head)^A,B^	Brushing hair	✓	✓
	Item 5 (Bringing hand to opposite shoulder)^B^	Itching/scratching	✓	✓
**Self-feeding**	Item 23 (Place forearms and/or hands on table)^A,B^	Eating independently	✓	✓
	Item 21 (Turning a ball over in hand)^A,B^	Picking up food when eating	✓	✓
	Item 16 (Extending elbow to touch a pencil)^A,B^	Picking food off a table without help	✓	✓
	Item 20 (Tearing a sheet of paper)^A^	Opening a wrapper/food packaging	✓	✓
	Item 13 (Maintain a seated position)^B^	Eating while seated	✓	✓
**Reaching, picking up and holding objects**	Item 17 (Picking up coins)^A,B^	Picking up and holding small items	✓	✓
	Item 9 (Sitting on the floor)^A,B^	Holding objects in a seated position	✓	✓
	Item 10 (Leaning towards a ball)^A,B^	Reaching an object	✓	✓
	Item 27 (Touching the floor while standing)^A,B^	Touching the floor to pick up something	✓	✓
	Item 32 (Squatting)^A,B^	Picking something up from the floor	✓	✓
**Physical activity**	Item 28 (Walking on heels)^A,B^	Walking	✓	✓
	Item 30 (Running)^A,B^	Exercising	✓	✓
	Item 31 (Hopping)^A,B^	Exercising/playing sport/hopping	✓	✓
	Item 29 (Walking on a line)^B^	Walking	✓	✓
	Item 26 (Standing on one foot)^B^	Taking a step/walking	✓	✓
**Writing and technology use**	Item 22 (Pointing at drawings)^A,B^	Using a phone or other device/touchscreen device	✓	✓
	Item 18 (Going around the edge of a CD)^A,B^	Using a touchscreen device	✓	✓
	Item 19 (Pick up pencil and draw loops)^A,B^	Writing/drawing with a pen	✓	✓
	Item 20 (Tearing a sheet of paper)^B^	Using your hands to tear a piece of paper	✓	✓
**Social contact/engagement**	Item 14 (Raise the head from the chest)^A,B^	Having a conversation/engaging with others	✓	✓
	Item 1 (Turning head)^A,B^	Looking around the room	✓	✓
	Item 2 (Lifting head)^B^	Looking around the room	✓	✓
**Toileting**	Item 25 (Stand without support)^B^	Using a toilet independently	✓	✓
	Item 12 (Sitting down on a chair from standing)^B^	Using a toilet independently	✓	✓
	Item 24 (Standing up from sitting on chair)^B^	Standing from sitting on toilet	✓	✓
**Performing work/school activities**	Item 13 (Maintain a seated position)^A^	Doing work/schoolwork while seated	✓	✓

^A^The item was most frequently associated with the ADL based on the qualitative interview data

^B^The item was most frequently associated with the ADL based on the survey data

^✓^The specific aspect of the ADL was reported in relation to the patient-friendly MFM32 item in the qualitative interviews and/or quantitative online survey

^●^The specific aspect of the ADL was not included as a response option in the quantitative online survey and was also not reported in the free-text response option by any respondents

*ADL* Activities of daily living; *MFM32* 32-item Motor Function Measure
- Figure 4. The number of participants selecting voice, difficulty sleeping, and other as important to maintain/improve has been corrected.

**Fig. 4:**
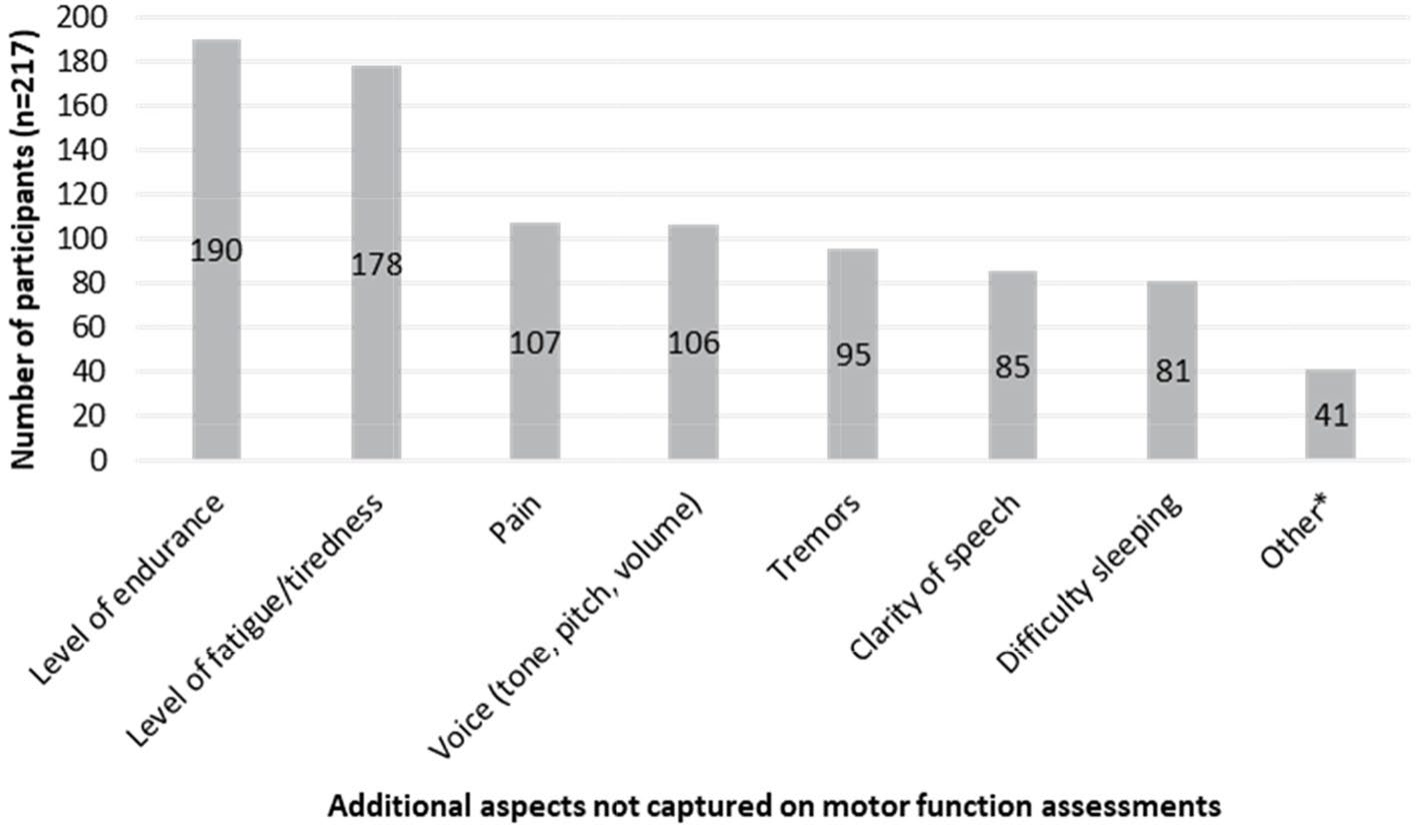
Additional symptoms and impacts that are important to maintain/improve not captured on motor function assessments. *Breathing/respiratory function (*n* = 12), muscle strength (*n* = 7), chewing/coughing/swallowing (*n* = 4), mental/psychological problems (*n* = 4), elimination of contractures/scoliosis (*n* = 3), general physical safety (*n* = 2), ability to feed oneself/brush teeth/sign name (*n* = 1), comfort (*n* = 1), exercise assessment (*n* = 1), losing weight (*n* = 1), moving position (*n* = 1), poor blood circulation/cold feet (*n* = 1), sexual life (*n* = 1), social interactions (*n* = 1), reason unclear (*n* = 1)

Additional information has been added in the Acknowledgements and Declarations sections:

- Carl Cooper (Adelphi Values) wrote the first draft of the manuscript.
- The online survey was conducted following market research principles.
- AB, TD and CV received consultancy fees from Roche for this project.
- LY, SR and ADM are employees of Charles River Associates commissioned by Roche to conduct the online survey.

The original article has been corrected. The main findings of the original article are not affected after this correction.
