# Supplementary material for: Understanding the relationship between the 32-item motor function measure and daily activities from an individual with spinal muscular atrophy and their caregivers’ perspective: a two-part study
Source: BMC Neurol. 2021 Mar 31;21:143. doi: 10.1186/s12883-021-02166-z (PMC8011105; doi:10.1186/s12883-021-02166-z)
Supplement: Supplementary file 1 — Additional file 1: Table S1. Detailed analysis of responses to the patient-friendly MFM32 items in quantitative survey. [file 12883_2021_2166_MOESM1_ESM.docx]

Supplementary Table 1. Detailed analysis of responses to the patient-friendly MFM32 items in quantitative survey

| **MFM32 item number** | **Clinician-reported MFM32 item**  **(maximum score)** | **MFM32 patient-friendly item** | **Number and proportion of participants presented with ability** | **Number and proportion of participants responding “can do”** | **Number and proportion of participants responding “cannot do”** | **Number and proportion of participants selecting at least one ADL via pre-defined list or via “other” response option** | **Number and proportion of participants that did not select any ADL or “other” response (non-response)** |
| --- | --- | --- | --- | --- | --- | --- | --- |
| **1** | Supine, hold head for 5 seconds in midline and turns completely from one side to another (D2) | When lying on your back, can you hold your head for 5 seconds and turn it from side to side? | 197 (91%) | 112 (57%) | 85 (43%) | 101 (90%) | 11 (10%) |
| **2** | Supine, raises head and maintains for 5 seconds (D2) | When lying on your back, can you lift your head and keep it lifted for 5 seconds? | 170 (78%) | 84 (49%) | 86 (51%) | 76 (90%) | 8 (10%) |
| **3** | Supine, flexes hip and knee more than 90 degrees by raising foot (D2) | When lying on your back, can you bring one knee to your chest? | 166 (76%) | 45 (27%) | 121 (73%) | 39 (87%) | 6 (13%) |
| **4** | Supine, leg supported, plantar flexion to dorsiflexion of foot to 90 degrees (D3) | When lying on your back, can you go from pointing your toes to flexing your foot? | 204 (94%) | 143 (70%) | 61 (30%) | 109 (76%) | 34 (24%) |
| **5** | Supine, raises hand and moves to the opposite shoulder (D2) | When lying on your back, can you bring one hand to the opposite shoulder? | 195 (90%) | 126 (65%) | 69 (35%) | 107 (85%) | 19 (15%) |
| **6** | Supine, legs half flexed, raises pelvis (D1) | When lying on your back, with your knees bent and your feet on the floor slightly apart, can you lift your hips up from the floor and hold for 5 seconds? | 152 (70%) | 43 (28%) | 109 (72%) | 37 (86%) | 6 (14%) |
| **7** | Supine to prone and frees upper limb from trunk (D2) | When lying on your back, can you turn over onto your stomach and free both of your arms? | 170 (78%) | 66 (39%) | 104 (61%) | 54 (82%) | 12 (18%) |
| **8** | Supine, sits up without upper limb support (D1) | When lying on your back, can you sit up without any extra help/support? | 107 (49%) | 32 (30%) | 75 (70%) | 30 (94%) | 2 (6%) |
| **9** | Seated on mat, maintains seated position for 5 seconds unsupported (D2) | When you are sitting, can you maintain a seated position and keep contact between the palms of your hands, without any extra help/support? | 185 (85%) | 141 (76%) | 44 (24%) | 122 (87%) | 19 (13%) |
| **10** | Seated on mat, leans forward to touch tennis ball (D2) | When you are sitting down, can you lean forward to touch an object, without any extra help/support? | 171 (79%) | 124 (73%) | 47 (27%) | 107 (86%) | 17 (14%) |
| **11** | Seated on mat, stands up without upper limb support (D1) | Can you stand up from sitting on the floor without any extra help/support? | 50 (23%) | 11 (22%) | 39 (78%) | 10 (91%) | 1 (9%) |
| **12** | Standing to sitting on chair without upper limb support (D1) | Can you sit down on a chair from standing without any extra help/support? | 55 (25%) | 40 (73%) | 15 (27%) | 39 (98%) | 1 (3%) |
| **13** | Seated on chair with no upper limb support for 5 seconds (D2) | Can you sit on a chair for 5 seconds with your head/body in the center? | 181 (83%) | 150 (83%) | 31 (17%) | 132 (88%) | 18 (12%) |
| **14** | Seated, from head in flexion, raises and maintains for 5 seconds in midline (D2) | When seated and looking at the floor, can you lift your head up and keep it lifted for 5 seconds? | 198 (91%) | 173 (87%) | 25 (13%) | 141 (82%) | 32 (18%) |
| **15** | Seated, forearms but not elbows on table, place both hands on top of head, with trunk and head remaining in midline (D2) | When you are sitting down, with your forearms but not elbows on the table, can you bring your arms up to put both hands on top of your head, without moving your body? | 189 (87%) | 81 (43%) | 108 (57%) | 68 (84%) | 13 (16%) |
| **16** | Seated on chair, without moving the trunk, reaches the pencil with one hand and forearms/hand off the table and elbow full extension (D2) | When you are sitting down with your forearm on the table, can you touch the pencil in front of you, without moving your body? | 199 (92%) | 155 (78%) | 44 (22%) | 130 (84%) | 25 (16%) |
| **17** | Pick up 10 coins (D3) | When you are sitting down with your forearm on the table, can you pick up 10 coins in your hand and hold them? | 217 (100%) | 160 (74%) | 57 (26%) | 132 (83%) | 28 (18%) |
| **18** | Seated, traces edge of CD without hand support on table (D3) | When you are sitting down with your forearm on the table, can you trace the edges of a circle using your finger? | 217 (100%) | 193 (89%) | 24 (11%) | 149 (77%) | 44 (23%) |
| **19** | Pick up the pencil and draw inside the frame (D3) | When you are sitting down with your elbow on or off the table, can you pick up the pencil in front of you and draw loops inside a box? | 204 (94%) | 181 (89%) | 23 (11%) | 142 (78%) | 39 (22%) |
| **20** | Tear sheet of paper (D3) | Can you tear a sheet of paper that has been folded in half and then in half again? | 174 (80%) | 118 (68%) | 56 (32%) | 103 (87%) | 15 (13%) |
| **21** | Seated, picks ball up and turns hand over completely (D3) | When you are sitting down with your forearm on the table in front of you, can you pick up the ball directly in front of you and turn your hand over? | 217 (100%) | 161 (74%) | 56 (26%) | 130 (81%) | 31 (19%) |
| **22** | Seated, raises finger and touches eight drawings successively without touching the lines (D3) | When you are sitting down, can you lift your finger and touch the drawings on a postcard in front of you without touching the red lines? | 50 (23%) | 45 (90%) | 5 (10%) | 45 (100%) | 0 (0%) |
| **23** | Seated, places two forearms on the table at the same time without moving trunk (D2) | When you are sitting down, starting with your hands by your sides, can you lift your arms to place both forearms/hands on the table at the same time while keeping your body still/stable? | 217 (100%) | 136 (63%) | 81 (37%) | 110 (81%) | 26 (19%) |
| **24** | Seated on chair, stands up without upper limb support (D1) | Starting seated on a chair, can you stand up without using your arms for support? | 52 (24%) | 11 (21%) | 41 (79%) | 11 (100%) | 0 (0%) |
| **25** | Standing with upper limb support for 5 seconds (D1) | Can you stand up without using your arms for support for 5 seconds? | 141 (65%) | 44 (31%) | 97 (69%) | 37 (84%) | 7 (16%) |
| **26** | Standing with upper limb support, raises foot for 10 seconds (D1) | When standing up, can you lift your foot from the floor for 10 seconds without support? | 87 (40%) | 37 (43%) | 50 (57%) | 30 (81%) | 7 (19%) |
| **27** | Standing, without support bends to touch the floor and stands up again (D1) | When standing, can you bend down to touch the floor and stand up again without any help/support? | 69 (32%) | 14 (20%) | 55 (80%) | 13 (93%) | 1 (7%) |
| **28** | Standing without support, takes 10 steps forward (D1) | Can you stand and take 10 steps forward on both heels? | 61 (28%) | 18 (30%) | 43 (70%) | 16 (89%) | 2 (11%) |
| **29** | Takes 10 steps forward on a line without support (D1) | Can you take 10 steps forward on a straight line without support? | 82 (38%) | 42 (51%) | 40 (49%) | 37 (88%) | 5 (12%) |
| **30** | Standing, runs 10 meters (D1) | Can you run 10 meters? | 45 (21%) | 7 (16%) | 38 (84%) | 7 (100%) | 0 (0%) |
| **31** | Standing on one foot without support, hops 10 times (D1) | Can you hop on one leg 10 times without help/support? | 45 (21%) | 3 (7%) | 42 (93%) | 3 (100%) | 0 (0%) |
| **32** | Squat without upper limb support (D1) | Can you squat (crouch or sit with knees bent) and then stand back up again twice in a row? | 43 (20%) | 7 (16%) | 36 (84%) | 7 (100%) | 0 (0%) |

Note: The items in the patient-friendly version of the MFM32 was also reordered based on prior Rasch measurement theory analyses by increasing level of difficulty. The total of the final two columns equates to the total number of participants who responded “can do” and were thus presented with the opportunity to provide an ADL considered to be relevant to the patient-friendly MFM32 items or not.
