# Supplementary material for: Understanding the relationship between the 32-item motor function measure and daily activities from an individual with spinal muscular atrophy and their caregivers’ perspective: a two-part study
Source: BMC Neurol. 2021 Mar 31;21:143. doi: 10.1186/s12883-021-02166-z (PMC8011105; doi:10.1186/s12883-021-02166-z)
Supplement: Supplementary file 2 — Additional file 2. Supplementary File 1. Qualitative interview guide for caregivers. [file 12883_2021_2166_MOESM2_ESM.docx]

Supplementary File 1. Qualitative interview guide for caregivers.

| **Understanding meaningful change on the Motor Function Measure (MFM-32) for individuals with Spinal Muscular Atrophy (SMA) using qualitative interviews** |
| --- |

# Interview objectives and overview

**Interview objectives and overview**

**[Note to interviewer]:**

The overall objective of this interview study is to explore how items included in the MFM-32 relate to activities of daily living from the perspective of caregivers and individuals with type II and type III SMA. This will be achieved by the conduct of qualitative interviews.

**Overview of the interview process**

The interview should be conducted as follows:

- **Part 1: Consent (5 minutes)**

The interviewer will introduce themselves and Adelphi Values to the participant. Explain the objectives and the interview process. Also obtain verbal consent for the interview to be audio-recorded.

- **Part 2: MFM-32 items and activities of daily living (40 minutes)**

Prior to the interview, participants will have completed a lay summary of the MFM-32 to give an approximate assessment of the current level of function of the individual they care for. This will help to inform which items are explored in more detail during the interview.

During this section of the interview, participants will be presented with lay summaries of the individual items of the MFM-32, relative to the level of functional ability. They will then be asked to comment on how the function/ability being assessed by each item relates to activities of daily living.

In total, the interview should take approximately **45-minutes**

**Instructions to Interviewer**

**The role of the interviewer before the interview**

- Check that the information provided on the patient and clinician screener aligns with the inclusion and exclusion criteria.
- Check the participant has provided written informed consent/assent to take part in the interview and for the interview to be audio-recorded.
- Check the participant has completed the demographic form.
- Check that the participant completed the MFM-32 motor function assessment within the demographics form and use the responses to tailor the interview according to the current level of motor functioning of the individual with SMA.

**The role of the interviewer during the interview**

- **Digital audio-recording:** Ensure the participant’s voice is clearly recorded throughout the interview. The audio-recording will be transcribed verbatim. Check the volume settings and positioning of the audio-recorder to ensure clarity of the recording prior to the interview. Avoid rustling papers near the recorder or jostling the recorder during the interview as this will lead to inaudible responses.
- **Interview guide:** Use this document as a guide, rather than a script. Where relevant, use your own wording when framing the questions (e.g. for younger participants) and follow the lead of the participant in terms of phrasing and terminology.

The role of the interviewer is to **inquire**, **support** and **listen**:

- Do not give your own point of view.
- Be patient and accept silence during open-ended questioning to make sure the individual has time to think about their response.
- Help the individual stick to the topics intended in the guide and avoid repeating him/herself.
- Summarize, reflect back and help the individual cross-reference and make connections that he/she would not do spontaneously.
- Ask the individual to clarify any comments or vague references that are relevant to the study aims.
- If the participant directs any questions to you that are of a medical nature, please explain that you are not a medical professional and that the participant should direct any medical queries to his or her doctor or nurse.
- **Most importantly:** avoid biasing or leading the individual. As much as possible your questions should be open-ended and general, rather than specific. You should avoid closed-questions, easily answered ‘yes’ or ‘no’. It is very important that you do not lead the participant with your questions or remarks or bias their responses. You should avoid using statements or questions that lead the participant or encourage social or treatment desirability or agreement bias in response to the wording or framing of the questions you pose. Allow the participant enough time to answer. Encourage participants to explain his or her responses to ensure a clear understanding of the intent of the participant. Possible non-leading probes that can be used to explore the participant’s perspective include:
- Can you describe exactly what that means for you?
- Can you explain why this would be an important change?
- Tell me more about that.
- How does that affect you?
- Can you talk more about ___________?

**Using the interview guide**

This guide is formatted so that it is clear which instructions are for the interviewer’s reference, and which instructions and questions are intended for the participant, and which of these are compulsory questions and which are probes (to be used where necessary). The formatting is as follows:

| **UPPER CASE TEXT** | These are guidance notes/instructions for you (the interviewer). These are in bold blue text. |
| --- | --- |
|  | These are statements and instructions that you read aloud to the participant. |
| 1. **Bold text** | Questions prefixed by a specific number and in bold text must be posed to all participants |
| - *Italic text* | All probes or follow up questions are bulleted – these do not necessarily need to be used, but are included to assist you in drawing further information from participants if needed. |

**Part 1: Consent (5 minutes)**

**Introductions**

**INTRODUCE YOUSELF AS WORKING FOR ADELPHI VALUES**

- Thank you for taking the time to talk to us today.
- My name is [first name]. I work for a company called Adelphi Values in Manchester, UK. Adelphi Values is a company that conducts health research that works with pharmaceutical companies to develop questionnaires for use in clinical studies to assess the impact of health conditions on people’s lives.

**EXPLAIN THE AIMS AND PROCESS OF THE INTERVIEW**

- During the interview today we will discuss the MFM-32. The MFM-32 is an assessment that is used by doctors to assess the physical abilities of individuals with SMA.
- The purpose of this research is to learn more about how the MFM-32 is related to physical abilities required to perform everyday activities.
- During the interview I will ask you to comment on how the physical abilities that are assessed in the MFM-32 relate to daily life. If you do not understand the ability being measured, please let me know and I will rephrase.
- This interview should last approximately 45 minutes.

**REASSURE THE PARTICIPANT OF CONFIDENTIALITY AND ANONYMITY**

- All information that you provide will be treated confidentially and will be anonymized by assigning you a unique ID number. Your name and contact information will remain with Adelphi Values and will only be accessible to the Adelphi Values’ project team who are conducting this study.
- If you report a bad experience (an adverse event) that the individual you care for has experienced as a direct result of taking a medication made by the study sponsor, Adelphi Values are required to report this to them. Any information you provide about the event will be treated in confidence and used solely for the purpose of drug safety monitoring.

**Audio-recording the interview**

- The interview today will be audio-recorded to enable me to pay careful attention to what you say and to make certain I accurately capture what you share with me during the interview. Please try to speak loudly and clearly so that your comments can be heard on the audio-recording.
- Please be honest in your responses and don’t be afraid to voice any opinions, there are no right or wrong answers to these questions.
- If you have any questions for me, you can ask them at any time.

**Verbal consent – turn on the audio-recorder**

**TURN ON THE AUDIO-RECORDER**

- This is [name of interviewer] with participant [participant ID number] on [date of interview] at [time of interview]. The interview is being conducted by Skype/telephone and I am located in XXXX [state location of interviewer including city and country e.g. London, UK], the person I am interviewing is in XXXX [state location of interviewee including city and country e.g. London, UK].
- Do you agree to participate in the interview?
- Do you agree to have this interview audio-recorded?
  - [IF PARTICIPANT DOES NOT AGREE TO HAVE THE INTERVIEW RECORDED: Thank the participant for taking part and end the interview and switch off the recorder.]
- Do you have any questions before we start?
- OK, I’ll start now, OK?

**Part 2: MFM-32 items and activities of daily living (40 minutes)**

**IN FRONT OF YOU PLEASE HAVE:**

- **The motor function assessment (included in the demographics form) that the participant completed before the interview.**
- **The lay summary of MFM-32 items and associated daily activities (to be used as probes; Appendix A (of this interview guide).**
  - **The items to be explored in the interview may be highlighted in advance by the interviewer to aid discussion.**
    - **For ambulant patients, focus on items that fall into D1 or D2 and for non-ambulant patients focus on items that fall into D2 and D3.**
- Before the interview you completed a form that asked you how well the individual you care for can perform particular physical abilities that are assessed by the MFM-32. We will focus this discussion on the ones that you said they could fully complete and then some that they can start but are not able to finish or can complete but with some help, slowly, without complete control or can’t hold for long, and how these relate to things the individual does in their daily life.
- Let’s start by discussing the first physical ability that you said the individual can fully complete.

1. **You said that the individual is “able to fully complete XXXX” [use lay summary language of MFM-32 item here].**
   - *Tell me about things that they do in their daily life that involve this movement/ability?*
     - *For example: [please use ADLs identified by experts for each item as a probe, if required]*
   - *Is this an important ability for the individual to be able to do? Why/why not?*

- **PLEASE CONTINUE TO ASK THE CAREGIVER ABOUT ABILITIES THEY HAVE INDICATED THE INDIVIDUAL CAN FULLY COMPLETE.**
- **IF TIME PERMITS, PLEASE ASK THE FOLLOWING QUESTION RELATING TO MFM-32 ITEMS THAT THE CAREGIVER REPORTED THE INDIVIDUAL IS ABLE TO START BUT IS NOT ABLE TO FINISH OR IS ABLE TO PERFORM BUT WITH SOME HELP, SLOWLY, WITHOUT COMPLETE CONTROL OR CAN’T HOLD FOR LONG.**

1. **You said that the individual is “able to start but is not able to finish XXXX” [use lay summary language of MFM-32 item here].**
   - *Tell me about how this affects things they do in their daily life?*
     - *For example: [please use ADLs identified by experts for each item as a probe, if required]*
   - *Is this an important ability for the individual to be able to do? Why/why not?*

- **PLEASE CONTINUE TO ASK THE CAREGIVER ABOUT ABILITIES THEY HAVE INDICATED THE INDIVIDUAL CAN START BUT IS NOT ABLE TO FINISH OR CAN PERFORM BUT WITH SOME HELP, SLOWLY, WITHOUT COMPLETE CONTROL OR CAN’T HOLD FOR LONG.**
- **IF TIME PERMITS, PLEASE ASK THE FOLLOWING QUESTION RELATING TO MFM-32 ITEMS THAT THE CAREGIVER REPORTED THE INDIVIDUAL IS UNABLE TO COMPLETE.**

1. **You said that the individual is “unable to complete XXXX” [use lay summary language of MFM-32 item here].**
   - *What things in their daily life are they unable to do or find difficult because they can’t do this?*
     - *For example: [please use ADLs identified by experts for each item as a probe, if required]*
   - *Is this an important ability for the individual to be able to do? Why/why not?*

**END OF INTERVIEW**

**Appendix A: MFM-32 items and lay summary and associated ADLs**

| MFM-32 item | Lay summary | ADLs (to be used as probes in initial interviews) |
| --- | --- | --- |
| 22. Seated, raises finger and touches 8 drawings successively without touching the lines (D3) | When you are sat down, can you lift your finger and touch the drawings in front of you without touching the lines? | - Use a computer - Use a touchscreen device - Use a phone or a control of environment |
| 18. Seated, traces edge of CD without hand support on table (D3) | When you are sat down with your forearm on the table, can you trace the edges of a CD | - Use a computer - Use a touchscreen device |
| 17. Pick up 10 coins (D3) | When you are sat down with your forearm on the table, can you pick up 10 coins in your hand in 20 seconds and hold them? | - Pick up and hold small items such as coins or keys or cutlery - Write with pen |
| 23. Seated, places two forearms on the table at the same time without moving trunk (D2) | When you are sat down, starting with your hands by your sides, can you lift your arms to place both forearms/hands on the table at the same time while keeping your body still? | - hold objects in a sitting position - Use the toilet independently |
| 21. Seated, picks ball up and turns hand over completely (D3) | When you are sat down with your forearm on the table, can you pick up a ball in front of you and turn your hand over? | - Turn pages of book |
| 19. Pick up pencil and draw inside the frame (D3) | When you are sat down with your elbow on or off the table, can you pick up a pencil and draw loops inside a frame? | - Write with pen |
| 4. Supine, leg supported, plantar flexion to dorsiflexion of foot to 90degrees (D3) | When lying on your back, can you go from pointing your toes to flexing your foot? | - Putting on shoes |
| 16. Seated on chair, without moving the trunk, reaches the pencil with one hand and forearms/hand off the table and elbow full extension (D2) | When you are sat down with your forearm on the table, can you touch a pencil in front of you, without moving your body? | - Reach an object at arm’s length, e.g. from a table - Pick up food off a table without help - Write with pen - Use a computer - Use a phone or a control of environment |
| 14. Seated, from head in flexion, raises and maintains for 5secs in midline (D2) | When seated on the floor, starting from looking at the floor, can you lift your head up and keep it lifted for 5 seconds? | - Head control when sitting |
| 1. Supine, hold head for 5secs in midline and turns completely from one side to another (D2) | When lying on your back, can you hold your head for 5 seconds and turn it from side to side? | - Head control when lying on your back |
| 5. Supine, raises hand and moves to the opposite shoulder (D2) | When lying on your back, can you bring one hand to the opposite shoulder? | - Ability to hug |
| 15. Seated, forearms but not elbows on table, place both hands on top of head, with trunk and head remaining in midline (D2) | When you are sat down, with your forearms but not elbows on the table, can you bring your arms up to put both hands on top of your head, without moving your body? | - Brush hair - Brush teeth - Wash face - Wash body - Dressing - Self-feed |
| 9. Seated on mat, maintains seated position for 5secs unsupported (D2) | When seated on the floor, can you maintain a seated position and keep contact between your hands, without any extra help/support? | - Maintain seated position without backrest - Hold objects in a sitting position, e.g. a book, tablet |
| 13. Seated on chair with no upper limb support for 5secs (D2) | Can you sit on a chair for 5 seconds with your head/body in the centre? | - Maintain seated position without backrest - Wash body - Transfer from/to wheelchair |
| 20. Tear sheet of paper (D3) | Can you tear a sheet of paper that has been folded in half and then in half again? | - Turn pages of book |
| 10. Seated on mat, leans forward to touch tennis ball (D2) | When you are sat down, can you lean forward to touch a tennis ball, without any extra help/support? | - Lean forward then lean back again (e.g., in the wheelchair) - Carry objects in a sitting position - Wash your body - Getting in and out of your wheelchair without help - Dressing |
| 2. Supine, raises the head and maintains for 5secs (D2) | When lying on your back, can you lift your head and keep it lifted for 5 seconds? | - Head control when lying on back |
| 7. Supine to prone and frees upper limb from trunk (D2) | When lying on your back, can you turn over onto your stomach and free both of your arms? | - Turn and move in bed to change position |
| 3. Supine, flexes hip and knee more than 90degrees by raising foot (D2) | When lying on your back, can you bring one knee to your chest? | - Dressing lower body |
| 6. Supine, legs half flexed, raises pelvis (D1) | When lying on your back, with your knees bent and your feet on the floor slightly apart, can you lift your pelvis up from the floor and hold for 5 seconds? | - Dressing |
| 25. Standing with upper limb support for 5secs (D1) | Can you stand up without using your arms for support for 5 seconds? | - Stand - Use toilet independently |
| 8. Supine, sits up without upper limb support | When lying on your back, can you sit up without any extra help/support? | - Sit up from lying on back |
| 26. Standing with upper limb support, raises the foot for 10 secs (D1) | When standing up, can you lift your foot from the floor for 10 seconds without support? | - Stand on one foot |
| 29. Takes 10 steps forward on a line without support (D1) | Can you take 10 steps forward on in a straight line without support? | - Walk |
| 27. Standing, without support bends to touch the floor and stands up again (D1) | When standing, can you bend down to touch the floor and stand up again without any help/support? | - Touch floor to pick something up |
| 28. Standing without support, takes 10 steps forward (D1) | Can you stand and take 10 steps forward on both heels? | - Walk |
| 12. Standing to sitting on chair without upper limb support (D1) | Can you sit down on a chair from standing without any extra help/support? | - Sit down from standing - Use toilet independently |
| 24. Seated on chair, stands up without upper limb support (D1) | Starting seated on a chair, can you stand up without using your arms for support? | - Stand from sitting on a chair - Transfer from/to wheelchair |
| 11. Seated on mat, stands up without upper limb support (D1) | Can you stand up from sitting on the floor without any extra help/support? | - Stand from sitting on the floor (for example after a fall) |
| 30. Standing, runs 10meters (D1) | Can you run 10 meters? | - Run |
| 31. Standing on one foot without support, hops 10 times (D1) | Can you hop on one leg 10 times without help/support? | - Hop |
| 32. Squat without upper limb support (D1) | Can you squat (crouch or sit with knees bent) and then stand back up again twice in a row? | - Do squats |
