# Supplementary material for: Understanding the relationship between the 32-item motor function measure and daily activities from an individual with spinal muscular atrophy and their caregivers’ perspective: a two-part study
Source: BMC Neurol. 2021 Mar 31;21:143. doi: 10.1186/s12883-021-02166-z (PMC8011105; doi:10.1186/s12883-021-02166-z)
Supplement: Supplementary file 4 — Additional file 4. Supplementary File 3. Online patient and caregiver survey. [file 12883_2021_2166_MOESM4_ESM.docx]

Supplementary File 3. Online patient and caregiver survey

**Motor Function Measure (MFM-32) Patient Survey**

[START OF SURVEY]

**Demographics**

1. Please indicate your gender:
   1. Male
   2. Female
   3. Prefer not to answer
2. Has your doctor told you that you have scoliosis?
   1. Yes (if YES, please indicate the Cobb angle if known)
      1. < 40-degree Cobb angle
      2. > 40-degree Cobb angle
      3. Unknown
   2. No
3. Do you have any contractures (i.e. do you feel you are unable to complete the full range of movement in any parts of your body)?
   1. Yes (if YES, please indicate where the contractures are present, select all that apply)
      1. Wrist
      2. Fingers
      3. Hip
      4. Knee
      5. Elbow
      6. Shoulder
      7. Ankle
      8. Forearm
   2. No
   3. Unknown
4. In your opinion, what percent of the time do you need assistance with day to day activities?
   1. 0 – 25% of the time
   2. 26 – 50% of the time
   3. 51 – 75% of the time
   4. 76 – 100% of the time

**Introductory Questions**

1. Thinking back to one year ago, please indicate if: [SELECT ONE]
   1. You have experienced some reduction in your ability to perform certain tasks
   2. Your ability to perform certain tasks has remained stable
   3. You have experienced some improvement in your ability to perform certain tasks
2. How do you expect your ability to perform certain tasks to change over the next year? [SELECT ONE]
   1. To experience some reduction in your ability to perform certain tasks
   2. To remain stable in your ability to perform certain tasks
   3. To experience some improvement in your ability to perform certain tasks

**Part 1 – Understanding the relevance of MFM-32 items to activities of daily living**

1. Please answer the questions below to the best of your knowledge, based on your opinion of your current level of functional ability. You do not need to attempt any of the tasks listed in order to answer.

[IF ‘CANNOT DO’ IS SELECTED FOR FIVE CONSECUTIVE ITEMS, STOP ASKING QUESTIONS AND SKIP TO PART 2]

|  | Ability | Can do (either partially or fully) | Cannot do | COLUMN A  Please select all of the daily activities from the list below that you think relate to this ability. Please detail any other activities if they are not listed. | COLUMN B  Importance of being able to do this ability in daily life |
| --- | --- | --- | --- | --- | --- |
| 22 | When you are sitting down, can you lift your finger and trace the border of a postcard in front of you? |  |  | - Using your finger to touch buttons on a mobile phone - Using your finger to work a touchscreen device (e.g. iPad) - Using your fingers to type on a keyboard/computer - Pointing to communicate - Reaching for objects - Other [FREE TEXT BOX] | - Important to be able to use technology (e.g. using a phone/ computer/ iPad) - Helpful skill in daily life - Assesses level of independence - Other [FREE TEXT BOX] |
| 18 | When you are sitting down with your forearm on the table, can you trace the edges of a circle using your finger? |  |  | - Using your finger to work a touchscreen device (e.g. iPad) - Using your fingers to type on a keyboard/ computer, writing/drawing - Using finger to guide reading - Other [FREE TEXT BOX] | - Important skill to be able to use technology (e.g. using a phone/ computer/ iPad) - Enjoyment of related activities (e.g. drawing) - Being able to engage with others - Other [FREE TEXT BOX] |
| 17 | When you are sitting down with your forearm on the table, can you pick up 10 coins in your hand and hold them? |  |  | - Pick up and hold small items with your hands such as:   - Keys   - Cutlery   - Food/a glass   - Pens/Pencils   - Hairclips/paperclips   - Medication - Writing with a pen - Other [FREE TEXT BOX] | - Helpful skill in daily life - Increased independence - Demonstrates fine motor skills - Other [FREE TEXT BOX] |
| 23 | When you are sitting down, starting with your hands by your sides, can you lift your arms to place both forearms/hands on the table at the same time while keeping your body still/stable? |  |  | - Eating independently (without using devices or help) - Moving hands up to desk at school/work - Playing - Completing schoolwork/paperwork - Writing/drawing - Using a computer (without using devices or help) - Other [FREE TEXT BOX] | - Helpful skill in daily life - Increased independence - Ability to self-feed - Other [FREE TEXT BOX] |
| 21 | When you are sitting down with your forearm on the table in front of you, can you pick up a ball directly in front of you and turn your hand over? |  |  | - Turn pages of book using your hands - Picking up food when eating (without help) - Using cutlery when eating e.g. spoon, fork - Picking up an object in front of you and turning it over - Picking up a phone - Other [FREE TEXT BOX] | - Helpful skill in daily life - Increased independence - Other [FREE TEXT BOX] |
| 19 | When you are sitting down with your elbow on or off the table, can you pick up a pencil in front of you and draw loops inside a box? |  |  | - Writing/drawing - Using your fingers to touch buttons on a phone - Using your fingers to work a touchscreen device (e.g. iPad) - Using cutlery when eating (e.g. a fork) (without a device or help) - Other [FREE TEXT BOX] | - Demonstrates fine motor skills - Helpful skill in daily life - Ability to self-feed - Other [FREE TEXT BOX] |
| 4 | When lying on your back, can you go from pointing your toes to flexing your foot? |  |  | - Pointing your toes to put shoes on - Exercising/stretching - Pointing your toes to put socks on - Pointing your toes to help put on pants/trousers | - Keeping joints loose allows range of motion - Increased independence - Other [FREE TEXT BOX] |
| 16 | When you are sitting down with your forearm on the table, can you touch a pencil in front of you, without moving your body? |  |  | - Picking up food off a table without help - Using a computer (without devices or help)   Reaching for an object at arm’s length, e.g. from a table   - Using your hand/arm to pick up glass/cup to drink - Other [FREE TEXT BOX] | - Helpful skill in daily life - Increased independence - Other [FREE TEXT BOX] |
| 14 | When seated and looking at the floor, can you lift your head up and keep it lifted for 5 seconds? |  |  | - Having conversation/engaging with others - Holding eye contact - Looking up (e.g. when in car/driving) - Watching TV - Looking up from notes to a teacher - Other [FREE TEXT BOX] | - Helpful skill in daily life - Increased independence - Allows engagement /eye contact with others - Other [FREE TEXT BOX] |
| 1 | When lying on your back, can you hold your head for 5 seconds and turn it from side to side? |  |  | - Looking around the room - Looking around (e.g. when in car/driving) - Adjusting position in bed - Getting dressed while lying down (e.g. pulling shirt over-head) - Other [FREE TEXT BOX] | - Helpful skill in daily life - Increased independence - Other [FREE TEXT BOX] |
| 5 | When lying on your back, can you bring one hand to the opposite shoulder? |  |  | - Dressing upper body (e.g. lift your arms to put on a t-shirt or jumper) - Itching/scratching - To help with showering/bathing upper body - Pulling bed covers over shoulder - Other [FREE TEXT BOX] | - Helpful skill in daily life - Increased independence - Other [FREE TEXT BOX] |
| 15 | When you are sitting down, with your forearms but not elbows on the table, can you bring your arms up to put both hand on top of your head, without moving your body? |  |  | - Brushing hair - Dressing - Washing/drying hair - Washing your face - Raising hand in class / to say hello - Other [FREE TEXT BOX] | - Important to be able to self-care - Increased independence - Other [FREE TEXT BOX] |
| 9 | When you are sitting, can you maintain a seated position and keep contact between the palms of your hands, without any extra help/support? |  |  | - Maintaining a seated position without using hands for support - Holding objects (e.g. a book, tablet) in your hands whilst sitting - Other [FREE TEXT BOX] | - Demonstrates core strength and balance - Allows option to sit in different positions e.g. out of wheelchair - Other [FREE TEXT BOX] |
| 13 | Can you sit on a chair for 5 seconds with your head/body in the centre? |  |  | - Maintaining seated position without backrest - Doing work/schoolwork while seated - Sitting in a chair/wheelchair - Eating while seated - Sitting on the toilet - Other [FREE TEXT BOX] | - Helpful skill in daily life - Other [FREE TEXT BOX] |
| 20 | Can you tear a sheet of paper that has been folded in half and then in half again? |  |  | - Using your hands to turn pages of book - Using your hands to open a package/mail - Using your hands to open a wrapper/food packaging - Using your hands to tear a piece of paper - Using your hands/fingers to fasten buttons/zip - Other [FREE TEXT BOX] | - Helpful skill in daily life - Increased independence - Other [FREE TEXT BOX] |
| 10 | When you are sitting, can you lean forward to touch an object, without any extra help/support? |  |  | - Dressing lower body (e.g. putting on trousers/pants/socks) - Reaching for an object - Washing lower body when bathing - Picking up an object that has been dropped - Other [FREE TEXT BOX] | - Helpful skill in daily life - Increased independence - Other [FREE TEXT BOX] |
| 2 | When lying on your back, can you lift your head and keep it lifted for 5 seconds? |  |  | - Getting dressed (e.g. pulling shirt over your head) - Getting out of bed - Looking around the room - Lifting head to move a pillow - Bathing/washing hair - Other [FREE TEXT BOX] | - Increased independence - Demonstrates strength in neck - Other [FREE TEXT BOX] |
| 7 | When lying on your back, can you turn over onto your stomach and free both of your arms? |  |  | - Turning and moving in bed to change position - Other [FREE TEXT BOX] | - Important for comfort in bed and quality of sleep - Increased independence - Other [FREE TEXT BOX] |
| 3 | When lying on your back, can you bring one knee to your chest? |  |  | - Dressing lower body (e.g. putting on trousers/pants/socks) - Exercising/stretching (e.g. during physical therapy) - Getting into a bathtub - Showering/washing - Climbing stairs - Other [FREE TEXT BOX] | - Helpful skill in daily life - Important for stretching and mobility - Other [FREE TEXT BOX] |
| 6 | When lying on your back, with your knees bent and your feet on the floor slightly apart, can you lift your hips up from the floor and hold for 5 seconds? |  |  | - Putting on pants/trousers - Exercising/stretching - Showering/bathing - Transferring - Other [FREE TEXT BOX] | - Increased independence - Helpful skill in daily life - Other [FREE TEXT BOX] |
| 25 | Can you stand up without using your arms for support for 5 seconds? |  |  | - Using a toilet independently - Standing to dress/wash - Other [FREE TEXT BOX] | - Increased independence - Helpful skill in daily life - Demonstrates balance |
| 8 | When lying on your back, can you sit up without any extra help/support? |  |  | - Sitting up from lying on back - To help getting out of bed - Other [FREE TEXT BOX] | - Increased independence - Helpful skill in daily life - Other [FREE TEXT BOX] |
| 26 | When standing up, can you lift your foot from the floor for 10 seconds without support? |  |  | - Dressing lower body (e.g. putting on trousers/pants/socks) - Taking a step/walking - Getting into a car - Taking a step up into a shop/café - Other [FREE TEXT BOX] | - Demonstrates balance - Helpful skill in daily life - Other [FREE TEXT BOX] |
| 29 | Can you take 10 steps forward on in a straight line without support? |  |  | - Walking - Other [FREE TEXT BOX] | - Increased independence - Helpful skill in daily life - Other [FREE TEXT BOX] |
| 27 | When standing, can you bend down to touch the floor and stand up again without any help/support? |  |  | - Touching the floor to pick something up or put something down - Bending down to put on/tying shoes/socks - Showering/bathing - Other [FREE TEXT BOX] | - Increased independence - Helpful skill in daily life - Demonstrates flexibility - Other [FREE TEXT BOX] |
| 28 | Can you stand and take 10 steps forward on both heels? |  |  | - Walking - Other [FREE TEXT BOX] | - Increased independence - Helpful skill in daily life - Other [FREE TEXT BOX] |
| 12 | Can you sit down on a chair from standing without any extra help/support? |  |  | - Using toilet independently - Eating at home/restaurant - Transferring - Other [FREE TEXT BOX] | - Increased independence - Helpful skill in daily life - Demonstrates balance and lower body strength - Other [FREE TEXT BOX] |
| 24 | Starting seated on a chair, can you stand up without using your arms for support? |  |  | - Transferring from/to wheelchair - Standing from sitting on toilet - Standing from sitting at dinner table - Standing from sitting when carrying objects - Other [FREE TEXT BOX] | - Increased independence - Helpful skill in daily life - Demonstrates balance and lower body strength - Other [FREE TEXT BOX] |
| 11 | Can you stand up from sitting on the floor without any extra help/support? |  |  | - Standing from sitting on the floor - Standing after a fall - Other [FREE TEXT BOX] | - Increased independence - Helpful skill in daily life - Demonstrates balance and lower body strength - Other [FREE TEXT BOX] |
| 30 | Can you run 10 meters? |  |  | - Running - Exercising - Being able to participate in sports activities - Crossing the street | - Able to move quickly in emergencies - Demonstrates independent mobility - Other [FREE TEXT BOX] |
| 31 | Can you hop on one leg 10 times without help/support? |  |  | - Hopping - Exercising - Going upstairs - Other [FREE TEXT BOX] | - Important for health and wellbeing - Increased independence - Other [FREE TEXT BOX] |
| 32 | Can you squat (crouch or sit with knees bent) and then stand back up again twice in a row? |  |  | - Picking something up from the floor - Dressing - Other [FREE TEXT BOX] | - Helpful skill in daily life - Other [FREE TEXT BOX] |

[FOR EACH ITEM WITH A SCORE OF ‘CAN DO’, COLUMN A WILL POP UP. RESPONDENT WILL BE ASKED TO SELECT ALL ACTIVITIES THAT ARE LINKED TO THAT INITIAL ABILITY (USE TEXT IN COLUMN A TO PROMPT). LIST OF DAILY ACTIVITIES WILL NOT APPEAR FOR ITEMS SCORED ‘CANNOT DO’]

**Part 2 – Understanding the clinical meaningfulness of changes on MFM-32**

**Stabilization**

1. If you were to maintain a similar level of function on the abilities assessed by MFM-32 as you indicated in Part 1 and maintained your current level of function over the next year, would that be impactful/meaningful to you?
   - - - 1. Yes [IF YES, POP UP NEXT PORTION OF QUESTION] Please explain what maintaining a similar level of function would mean in terms of your daily life by selecting all the relevant options below.

- Maintain level of independence in daily life
- Maintaining current quality of life (emotional, social)
- Reassurance that my SMA condition will not progress
- Able to continue to perform certain tasks for the same length of time
- Other, please specify
  - - - 1. No [IF NO, POP UP THE OE QUESTION] Why not? [FREE TEXT RESPONSE]
        2. I don’t know

**Improvement in abilities currently able to perform**

1. For the abilities you previously responded that you can do, if you were able to improve your abilities by some level, would this represent an impactful/meaningful change in your life?
   - - - 1. Yes

[IF YES, POP UP NEXT PORTION OF QUESTION] Please explain what this improvement would mean in terms of your daily life by selecting all the relevant options below.

- Maintain level of independence in daily life
- Maintaining current quality of life (emotional, social)
- Other, please specify
  - - - 1. No

[IF NO, POP UP THE OE QUESTION] Why not? [FREE TEXT RESPONSE]

- - - - 1. I don’t know

**Improvement in abilities currently unable to perform**

[IDENTIFY FIRST THREE ITEMS SCORED AS ‘CANNOT DO’ IN PART 1]

[PRESENT FIRST ITEM SCORED AS ‘CANNOT DO’ IN PART 1]

For the question listed above, you previously responded that you are unable to perform the ability.

1. If you were to gain some level of ability to perform this task, would this represent an impactful/meaningful change in your life?
   - - - 1. Yes

[IF YES, POP UP NEXT PORTION OF QUESTION] Please explain what this change would mean in terms of your daily life by selecting all the relevant options below.

[FOR EACH ITEM PULL RESPONSE OPTIONS FROM COLUMN B (IMPORTANCE TO DAILY LIFE) AND PROVIDE LIST OF OPTIONS PLUS ‘OTHER’ OPTION]

- - - - 1. No

[IF NO, POP UP THE OE QUESTION] Why not? [FREE TEXT RESPONSE]

- - - - 1. I don’t know

[REPEAT FOR NEXT TWO ITEMS SCORED ‘CANNOT DO’ IN PART 1 UNTIL ALL THREE HAVE BEEN ASKED]

**Other abilities not captured by MFM-32 that would be important to maintain or improve**

1. What other aspects of your SMA would be important to maintain a current level of ability/functioning or improve in that are not captured on functional assessments such as the MFM-32? Please select all that apply from the list below.

- Level of fatigue/lack of energy (tiredness)
- Level of endurance (ability to carry out tasks for longer)
- Voice (tone, pitch, volume)
- Clarity of speech
- Difficulty sleeping
- Pain
- Tremors
- Other, please specify

**THANK YOU FOR YOUR PARTICIPATION**

[UK ONLY] We can now reveal the sponsor of this study. The sponsor of this study is Roche.

**Motor Function Measure (MFM-32) Caregiver Survey**

[START OF SURVEY]

**Demographics**

1. Do you provide care for more than one individual with Spinal Muscular Atrophy (SMA)?
   1. Yes [ASK 1A]
   2. No

1A) For the following questions, please only think about one individual with SMA that you care for

1. Please indicate the gender of the person for which you are caring:
   1. Male
   2. Female
   3. Prefer not to answer
2. Has the doctor of the person you are caring for told you/them that they have scoliosis?
   1. Yes (if YES, please indicate the Cobb angle if known)
      1. < 40-degree Cobb angle
      2. > 40-degree Cobb angle
      3. Unknown
   2. No
3. Does the person you are caring for have any contractures (i.e. do they feel they are unable to complete the full range of movement in any parts of their body)?
   1. Yes (if YES, please indicate where the contractures are present, select all that apply)
      1. Wrist
      2. Fingers
      3. Hip
      4. Knee
      5. Elbow
      6. Shoulder
      7. Ankle
      8. Forearm
   2. No
   3. Unknown
4. In your opinion, what percent of the time does your patient need assistance with day to day activities?
   1. 0 – 25% of the time
   2. 26 – 50% of the time
   3. 51 – 75% of the time
   4. 76 – 100% of the time
5. How many hours per week do you provide care to / assist the individual with SMA?
   1. Approximately ______ hours per week

**Introductory questions**

1. Thinking back to one year ago, please indicate if: [SELECT ONE]
   1. The individual with SMA has experienced some reduction in their ability to perform certain tasks
   2. Their ability to perform certain tasks has remained stable
   3. The individual with SMA has experienced some improvement in their ability to perform certain tasks
2. How do you expect the individual with SMA’s ability to perform certain tasks to change over the next year? [SELECT ONE]
   1. To experience some reduction in their ability to perform certain tasks
   2. To remain stable in their ability to perform certain tasks
   3. To experience some improvement in their ability to perform certain tasks

**Part 1 – Understanding the relevance of MFM-32 items to activities of daily living**

1. Please answer the questions below to the best of your knowledge, based on your opinion of the individual with SMA’s current level of functional ability. The individual does not need to attempt any of the tasks listed in order to answer. Please note that the tasks are written to address the patient directly, but please answer to the best of your ability for the individual.

[IF ‘CANNOT DO’ IS SELECTED FOR FIVE CONSECUTIVE ITEMS, STOP ASKING QUESTIONS AND SKIP TO PART 2]

|  | Ability | Can do (either partially or fully) | Cannot do | COLUMN A  Please select all of the daily activities from the list below that you think relate to this ability. Please detail any other activities if they are not listed. | COLUMN B  Importance of being able to do this ability in daily life |
| --- | --- | --- | --- | --- | --- |
| 22 | When you are sitting down, can you lift your finger and trace the border of a postcard in front of you? |  |  | - Using your finger to touch buttons on a mobile phone - Using your finger to work a touchscreen device (e.g. iPad) - Using your fingers to type on a keyboard/computer - Pointing to communicate - Reaching for objects - Other [FREE TEXT BOX] | - Important to be able to use technology (e.g. using a phone/ computer/ iPad) - Helpful skill in daily life - Assesses level of independence - Other [FREE TEXT BOX] |
| 18 | When you are sitting down with your forearm on the table, can you trace the edges of a circle using your finger? |  |  | - Using your finger to work a touchscreen device (e.g. iPad) - Using your fingers to type on a keyboard/ computer, writing/drawing - Using finger to guide reading - Other [FREE TEXT BOX] | - Important skill to be able to use technology (e.g. using a phone/ computer/ iPad) - Enjoyment of related activities (e.g. drawing) - Being able to engage with others - Other [FREE TEXT BOX] |
| 17 | When you are sitting down with your forearm on the table, can you pick up 10 coins in your hand and hold them? |  |  | - Pick up and hold small items with your hands such as:   - Keys   - Cutlery   - Food/a glass   - Pens/Pencils   - Hairclips/paperclips   - Medication - Writing with a pen - Other [FREE TEXT BOX] | - Helpful skill in daily life - Increased independence - Demonstrates fine motor skills - Other [FREE TEXT BOX] |
| 23 | When you are sitting down, starting with your hands by your sides, can you lift your arms to place both forearms/hands on the table at the same time while keeping your body still/stable? |  |  | - Eating independently (without using devices or help) - Moving hands up to desk at school/work - Playing - Completing schoolwork/paperwork - Writing/drawing - Using a computer (without using devices or help) - Other [FREE TEXT BOX] | - Helpful skill in daily life - Increased independence - Ability to self-feed - Other [FREE TEXT BOX] |
| 21 | When you are sitting down with your forearm on the table in front of you, can you pick up a ball directly in front of you and turn your hand over? |  |  | - Turn pages of book using your hands - Picking up food when eating (without help) - Using cutlery when eating e.g. spoon, fork - Picking up an object in front of you and turning it over - Picking up a phone - Other [FREE TEXT BOX] | - Helpful skill in daily life - Increased independence - Other [FREE TEXT BOX] |
| 19 | When you are sitting down with your elbow on or off the table, can you pick up a pencil in front of you and draw loops inside a box? |  |  | - Writing/drawing - Using your fingers to touch buttons on a phone - Using your fingers to work a touchscreen device (e.g. iPad) - Using cutlery when eating (e.g. a fork) (without a device or help) - Other [FREE TEXT BOX] | - Demonstrates fine motor skills - Helpful skill in daily life - Ability to self-feed - Other [FREE TEXT BOX] |
| 4 | When lying on your back, can you go from pointing your toes to flexing your foot? |  |  | - Pointing your toes to put shoes on - Exercising/stretching - Pointing your toes to put socks on - Pointing your toes to help put on pants/trousers - Other [FREE TEXT BOX] | - Keeping joints loose allows range of motion - Increased independence - Other [FREE TEXT BOX] |
| 16 | When you are sitting down with your forearm on the table, can you touch a pencil in front of you, without moving your body? |  |  | - Picking up food off a table without help - Using a computer (without devices or help)   Reaching for an object at arm’s length, e.g. from a table   - Using your hand/arm to pick up glass/cup to drink - Other [FREE TEXT BOX] | - Helpful skill in daily life - Increased independence - Other [FREE TEXT BOX] |
| 14 | When seated and looking at the floor, can you lift your head up and keep it lifted for 5 seconds? |  |  | - Having conversation/engaging with others - Holding eye contact - Looking up (e.g. when in car/driving) - Watching TV - Looking up from notes to a teacher - Other [FREE TEXT BOX] | - Helpful skill in daily life - Increased independence - Allows engagement /eye contact with others - Other [FREE TEXT BOX] |
| 1 | When lying on your back, can you hold your head for 5 seconds and turn it from side to side? |  |  | - Looking around the room - Looking around (e.g. when in car/driving) - Adjusting position in bed - Getting dressed while lying down (e.g. pulling shirt over-head) - Other [FREE TEXT BOX] | - Helpful skill in daily life - Increased independence - Other [FREE TEXT BOX] |
| 5 | When lying on your back, can you bring one hand to the opposite shoulder? |  |  | - Dressing upper body (e.g. lift your arms to put on a t-shirt or jumper) - Itching/scratching - To help with showering/bathing upper body - Pulling bed covers over shoulder - Other [FREE TEXT BOX] | - Helpful skill in daily life - Increased independence - Other [FREE TEXT BOX] |
| 15 | When you are sitting down, with your forearms but not elbows on the table, can you bring your arms up to put both hand on top of your head, without moving your body? |  |  | - Brushing hair - Dressing - Washing/drying hair - Washing your face - Raising hand in class / to say hello - Other [FREE TEXT BOX] | - Important to be able to self-care - Increased independence - Other [FREE TEXT BOX] |
| 9 | When you are sitting, can you maintain a seated position and keep contact between the palms of your hands, without any extra help/support? |  |  | - Maintaining a seated position without using hands for support - Holding objects (e.g. a book, tablet) in your hands whilst sitting - Other [FREE TEXT BOX] | - Demonstrates core strength and balance - Allows option to sit in different positions e.g. out of wheelchair - Other [FREE TEXT BOX] |
| 13 | Can you sit on a chair for 5 seconds with your head/body in the centre? |  |  | - Maintaining seated position without backrest - Doing work/schoolwork while seated - Sitting in a chair/wheelchair - Eating while seated - Sitting on the toilet - Other [FREE TEXT BOX] | - Helpful skill in daily life - Other [FREE TEXT BOX] |
| 20 | Can you tear a sheet of paper that has been folded in half and then in half again? |  |  | - Using your hands to turn pages of book - Using your hands to open a package/mail - Using your hands to open a wrapper/food packaging - Using your hands to tear a piece of paper - Using your hands/fingers to fasten buttons/zip - Other [FREE TEXT BOX] | - Helpful skill in daily life - Increased independence - Other [FREE TEXT BOX] |
| 10 | When you are sitting, can you lean forward to touch an object, without any extra help/support? |  |  | - Dressing lower body (e.g. putting on trousers/pants/socks) - Reaching for an object - Washing lower body when bathing - Picking up an object that has been dropped - Other [FREE TEXT BOX] | - Helpful skill in daily life - Increased independence - Other [FREE TEXT BOX] |
| 2 | When lying on your back, can you lift your head and keep it lifted for 5 seconds? |  |  | - Getting dressed (e.g. pulling shirt over your head) - Getting out of bed - Looking around the room - Lifting head to move a pillow - Bathing/washing hair - Other [FREE TEXT BOX] | - Increased independence - Demonstrates strength in neck - Other [FREE TEXT BOX] |
| 7 | When lying on your back, can you turn over onto your stomach and free both of your arms? |  |  | - Turning and moving in bed to change position - Other [FREE TEXT BOX] | - Important for comfort in bed and quality of sleep - Increased independence - Other [FREE TEXT BOX] |
| 3 | When lying on your back, can you bring one knee to your chest? |  |  | - Dressing lower body (e.g. putting on trousers/pants/socks) - Exercising/stretching (e.g. during physical therapy) - Getting into a bathtub - Showering/washing - Climbing stairs - Other [FREE TEXT BOX] | - Helpful skill in daily life - Important for stretching and mobility - Other [FREE TEXT BOX] |
| 6 | When lying on your back, with your knees bent and your feet on the floor slightly apart, can you lift your hips up from the floor and hold for 5 seconds? |  |  | - Putting on pants/trousers - Exercising/stretching - Showering/bathing - Transferring - Other [FREE TEXT BOX] | - Increased independence - Helpful skill in daily life - Other [FREE TEXT BOX] |
| 25 | Can you stand up without using your arms for support for 5 seconds? |  |  | - Using a toilet independently - Standing to dress/wash - Other [FREE TEXT BOX] | - Increased independence - Helpful skill in daily life - Demonstrates balance - Other [FREE TEXT BOX] |
| 8 | When lying on your back, can you sit up without any extra help/support? |  |  | - Sitting up from lying on back - To help getting out of bed - Other [FREE TEXT BOX] | - Increased independence - Helpful skill in daily life - Other [FREE TEXT BOX] |
| 26 | When standing up, can you lift your foot from the floor for 10 seconds without support? |  |  | - Dressing lower body (e.g. putting on trousers/pants/socks) - Taking a step/walking - Getting into a car - Taking a step up into a shop/café - Other [FREE TEXT BOX] | - Demonstrates balance - Helpful skill in daily life - Other [FREE TEXT BOX] |
| 29 | Can you take 10 steps forward on in a straight line without support? |  |  | - Walking - Other [FREE TEXT BOX] | - Increased independence - Helpful skill in daily life - Other [FREE TEXT BOX] |
| 27 | When standing, can you bend down to touch the floor and stand up again without any help/support? |  |  | - Touching the floor to pick something up or put something down - Bending down to put on/tying shoes/socks - Showering/bathing - Other [FREE TEXT BOX] | - Increased independence - Helpful skill in daily life - Demonstrates flexibility - Other [FREE TEXT BOX] |
| 28 | Can you stand and take 10 steps forward on both heels? |  |  | - Walking - Other [FREE TEXT BOX] | - Increased independence - Helpful skill in daily life - Other [FREE TEXT BOX] |
| 12 | Can you sit down on a chair from standing without any extra help/support? |  |  | - Using toilet independently - Eating at home/restaurant - Transferring - Other [FREE TEXT BOX] | - Increased independence - Helpful skill in daily life - Demonstrates balance and lower body strength - Other [FREE TEXT BOX] |
| 24 | Starting seated on a chair, can you stand up without using your arms for support? |  |  | - Transferring from/to wheelchair - Standing from sitting on toilet - Standing from sitting at dinner table - Standing from sitting when carrying objects - Other [FREE TEXT BOX] | - Increased independence - Helpful skill in daily life - Demonstrates balance and lower body strength - Other [FREE TEXT BOX] |
| 11 | Can you stand up from sitting on the floor without any extra help/support? |  |  | - Standing from sitting on the floor - Standing after a fall - Other [FREE TEXT BOX] | - Increased independence - Helpful skill in daily life - Demonstrates balance and lower body strength - Other [FREE TEXT BOX] |
| 30 | Can you run 10 meters? |  |  | - Running - Exercising - Being able to participate in sports activities - Crossing the street - Other [FREE TEXT BOX] | - Able to move quickly in emergencies - Demonstrates independent mobility - Other [FREE TEXT BOX] |
| 31 | Can you hop on one leg 10 times without help/support? |  |  | - Hopping - Exercising - Going upstairs - Other [FREE TEXT BOX] | - Important for health and wellbeing - Increased independence - Other [FREE TEXT BOX] |
| 32 | Can you squat (crouch or sit with knees bent) and then stand back up again twice in a row? |  |  | - Picking something up from the floor - Dressing - Other [FREE TEXT BOX] | - Helpful skill in daily life - Other [FREE TEXT BOX] |

[FOR EACH ITEM WITH A SCORE OF ‘CAN DO’, COLUMN A WILL POP UP. RESPONDENT WILL BE ASKED TO SELECT ALL ACTIVITIES THAT ARE LINKED TO THAT INITIAL ABILITY (USE TEXT IN COLUMN A TO PROMPT). LIST OF DAILY ACTIVITIES WILL NOT APPEAR FOR ITEMS SCORED ‘CANNOT DO’]

**Part 2 – Understanding the clinical meaningfulness of changes on MFM-32**

**Stabilization**

1. If the individual with SMA were to maintain a similar level of function on the abilities assessed by MFM-32 as you indicated in Part 1 and maintained their current level of function over the next year, would that be impactful/meaningful to them?
   - - - 1. Yes [IF YES, POP UP NEXT PORTION OF QUESTION] Please explain what maintaining a similar level of function would mean in terms of their daily life by selecting all the relevant options below.

- Maintain level of independence in daily life
- Maintaining current quality of life (emotional, social)
- Reassurance that their SMA condition will not progress
- Able to continue to perform certain tasks for the same length of time
- Other, please specify
  - - - 1. No [IF NO, POP UP THE OE QUESTION] Why not? [FREE TEXT RESPONSE]
        2. I don’t know

**Improvement in abilities currently able to perform**

1. For the abilities you previously responded that the individual with SMA can do, if they were able to improve their abilities by some level, would this represent an impactful/meaningful change in their life?
   - - - 1. Yes

[IF YES, POP UP NEXT PORTION OF QUESTION] Please explain what this improvement would mean in terms of the individual’s daily life by selecting all the relevant options below.

- Maintain level of independence in daily life
- Maintaining current quality of life (emotional, social)
- Other, please specify
  - - - 1. No

[IF NO, POP UP THE OE QUESTION] Why not? [FREE TEXT RESPONSE]

- - - - 1. I don’t know

**Improvement in abilities currently unable to perform**

[IDENTIFY FIRST THREE ITEMS SCORED AS ‘CANNOT DO’ IN PART 1]

[PRESENT FIRST ITEM SCORED AS ‘CANNOT DO’ IN PART 1]

For the question listed above, you previously responded that the individual with SMA is unable to perform the ability.

1. If the individual with SMA were to gain some level of ability to perform this task, would this represent an impactful/meaningful change in their life?
   - - - 1. Yes

[IF YES, POP UP NEXT PORTION OF QUESTION] Please explain what this change would mean in terms of their daily life by selecting all the relevant options below.

[FOR EACH ITEM PULL RESPONSE OPTIONS FROM COLUMN B (IMPORTANCE TO DAILY LIFE) AND PROVIDE LIST OF OPTIONS PLUS ‘OTHER’ OPTION]

- - - - 1. No

[IF NO, POP UP THE OE QUESTION] Why not? [FREE TEXT RESPONSE]

- - - - 1. I don’t know

[REPEAT FOR NEXT TWO ITEMS SCORED ‘CANNOT DO’ IN PART 1 UNTIL ALL THREE HAVE BEEN ASKED]

**Other abilities not captured by MFM-32 that would be important to maintain or improve**

1. What other aspects of the individual with SMA would be important to maintain a current level of ability/functioning or improve in that are not captured on functional assessments such as the MFM-32? Please select all that apply from the list below.

- Level of fatigue/lack of energy (tiredness)
- Level of endurance (ability to carry out tasks for longer)
- Voice (tone, pitch, volume)
- Clarity of speech
- Difficulty sleeping
- Pain
- Tremors
- Other, please specify

**THANK YOU FOR YOUR PARTICIPATION**

[UK ONLY] We can now reveal the sponsor of this study. The sponsor of this study is Roche.
